# Supplementary material for: Quantifying tumor specificity using Bayesian probabilistic modeling for drug and immunotherapeutic target discovery
Source: Cell Rep Methods. 2024 Nov 7;4(11):100900. doi: 10.1016/j.crmeth.2024.100900 (PMC11705768; doi:10.1016/j.crmeth.2024.100900)
Supplement: Document S1. Figures S1–S5 [file mmc1.pdf]

Cell Reports Methods, Volume *4*

Supplemental information

Quantifying tumor specificity using Bayesian probabilistic modeling for drug and immunotherapeutic target discovery

Guangyuan Li, Daniel Schnell, Anukana Bhattacharjee, Mark Yarmarkovich, and Nathan Salomonis

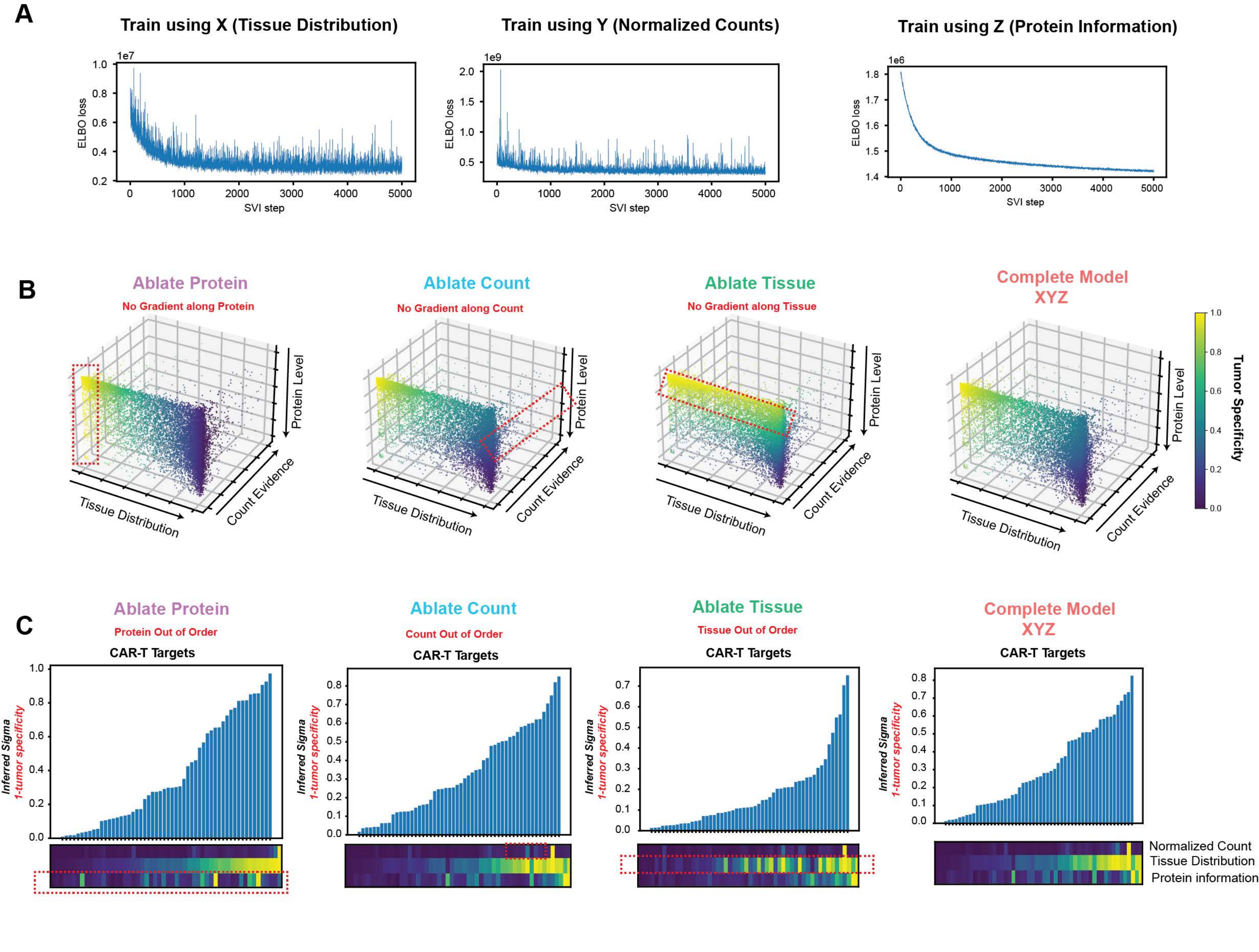

Supplementary Figure 1. BayesTS incorporates multiple data dimensions for inference. A) The Evidence Lower Bound (ELBO) loss across the training process when only one evidence is provided. B,C) Ablation test on each evidence and the exhaustive combinations of all forms of evidence for all 13,306 protein coding genes (B) and 54 CAR-T targets in clinical trials, the color corresponding to tumor specificity value, where higher values means highly tumor-specific or absent in normal tissue, and vice versa C) From left to right are X and Y, X and Z, Y and Z, X, Y and Z. Red box highlights the impact when each modality is left out. Y-axis indicates the BayesTS inferred sigma, which is the complement of tumor specificity score shown in B (1-sigma). Related to Figure 1 and 2.

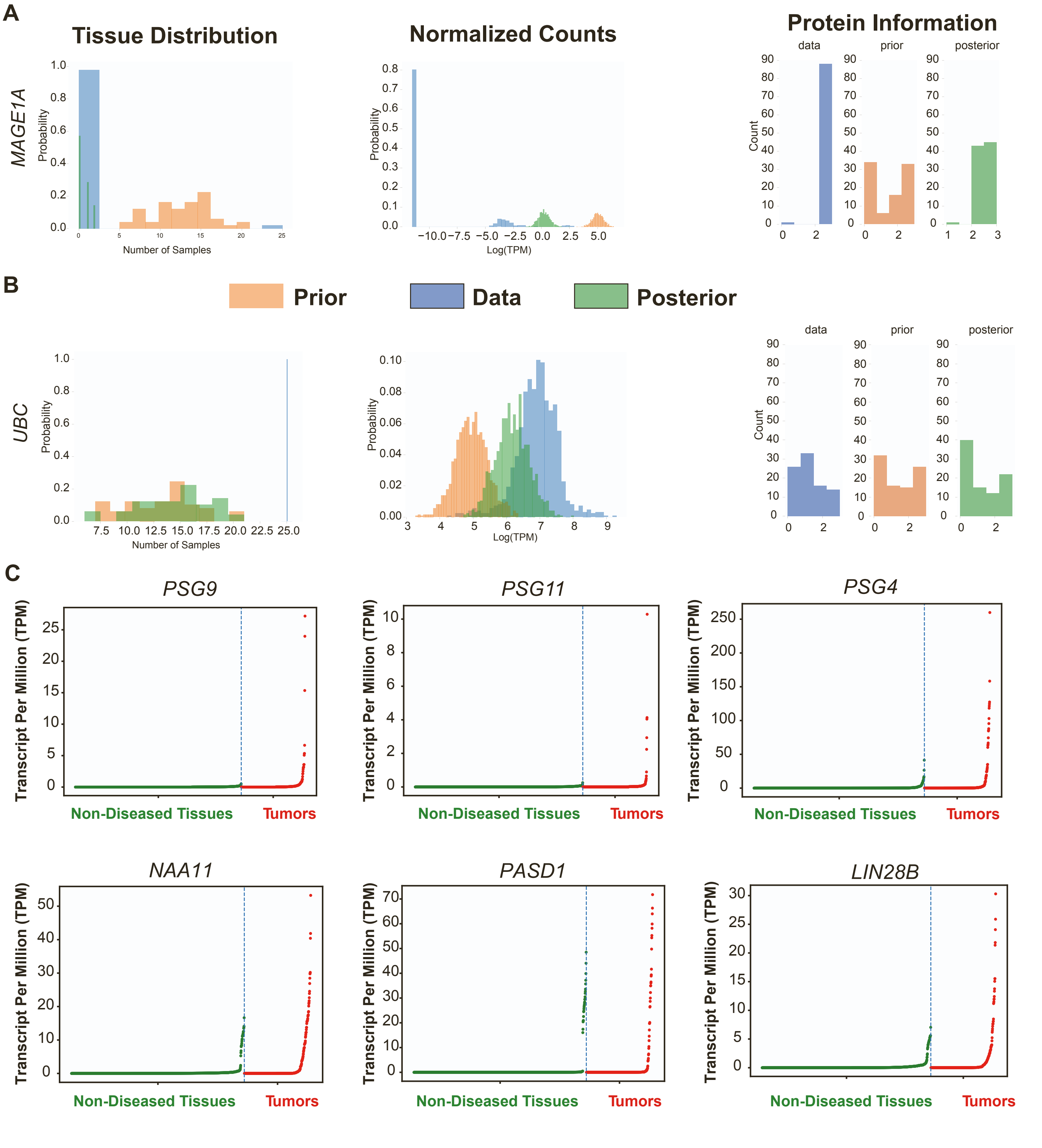

Supplementary Figure 2. Prior and Posterior check for the BayesTS inference process for two representative targets, along with novel drug targets using BayesTS A) A lowly expressed target, MAGE1A. B) A highly expressed target, UBC. From left to right, each panel represents one form of evidence, namely, tissue distribution X, normalized RNA count Y and protein-level information Z. Orange represents the prior distribution, blue the observed actual information and green the posterior distribution after the Bayesian variational inference. (C) Tissue-specificity RNA expression plots for control tissues (green) and melanoma patient samples (red), where each point is an independent sample (blue lines separating normal and tumor tissue samples, organized by tissue type). Examples are shown for Pregnancy-specific glycoproteins (PSG), namely PSG6, PSG9 and PSG4 and other potentially novel targets for therapy. Related to Figure 2 and 3.

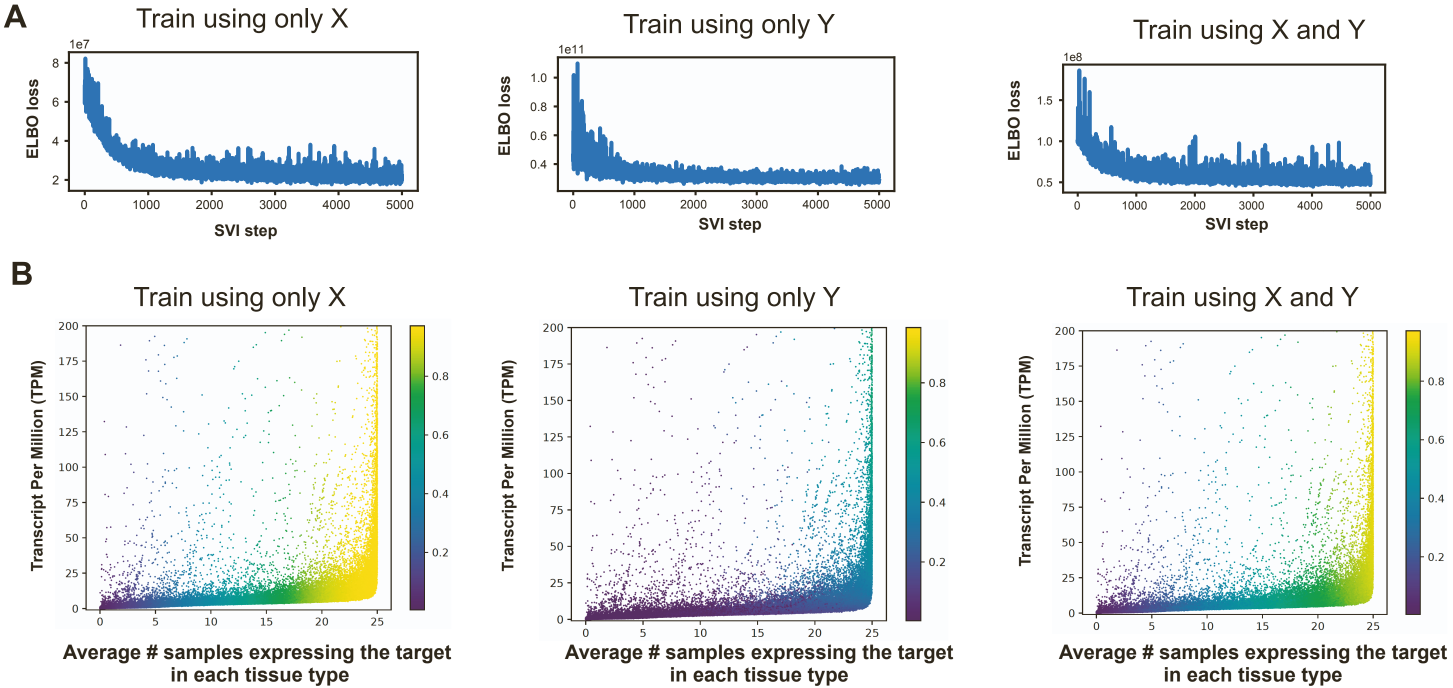

Supplementary Figure 3. Training loss and validation of BayesTS on splice junction targets. A) The ELBO loss of training tissue distribution (X) or normalized RNA count (Y) separately and together (right). B) The inferred tumor specificity scores projected on both X and Y axis in three different scenarios. Related to Figure 3.

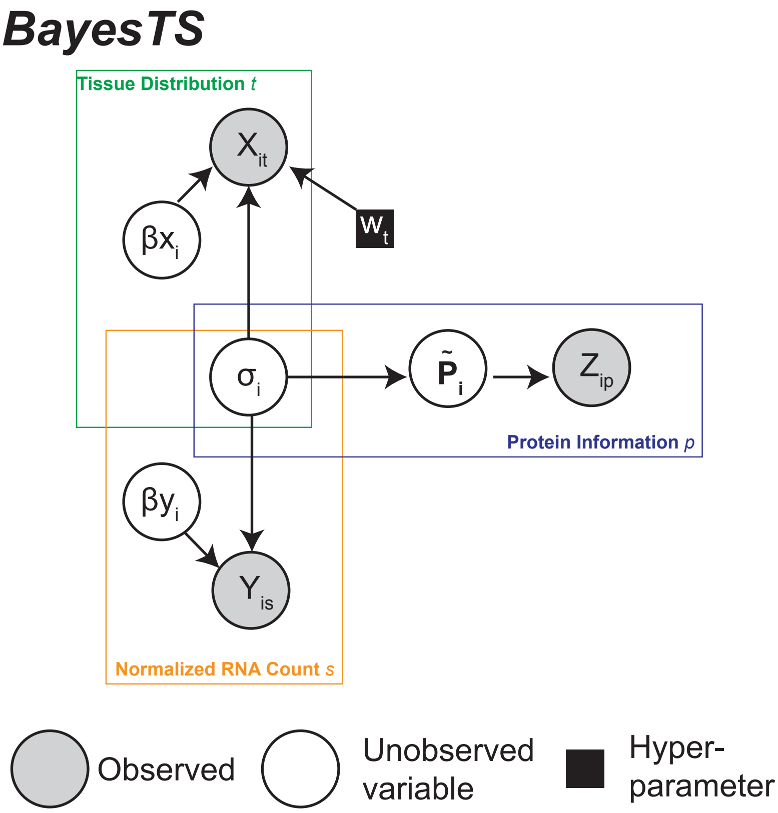

Supplementary Figure 4. The plate notation of the BayesTS model. Each circle represents a random variable in the model shade denotes the observed values and hollow denotes the unobserved values. Black squares represent tunable hyper-parameters. Related to STAR Method.

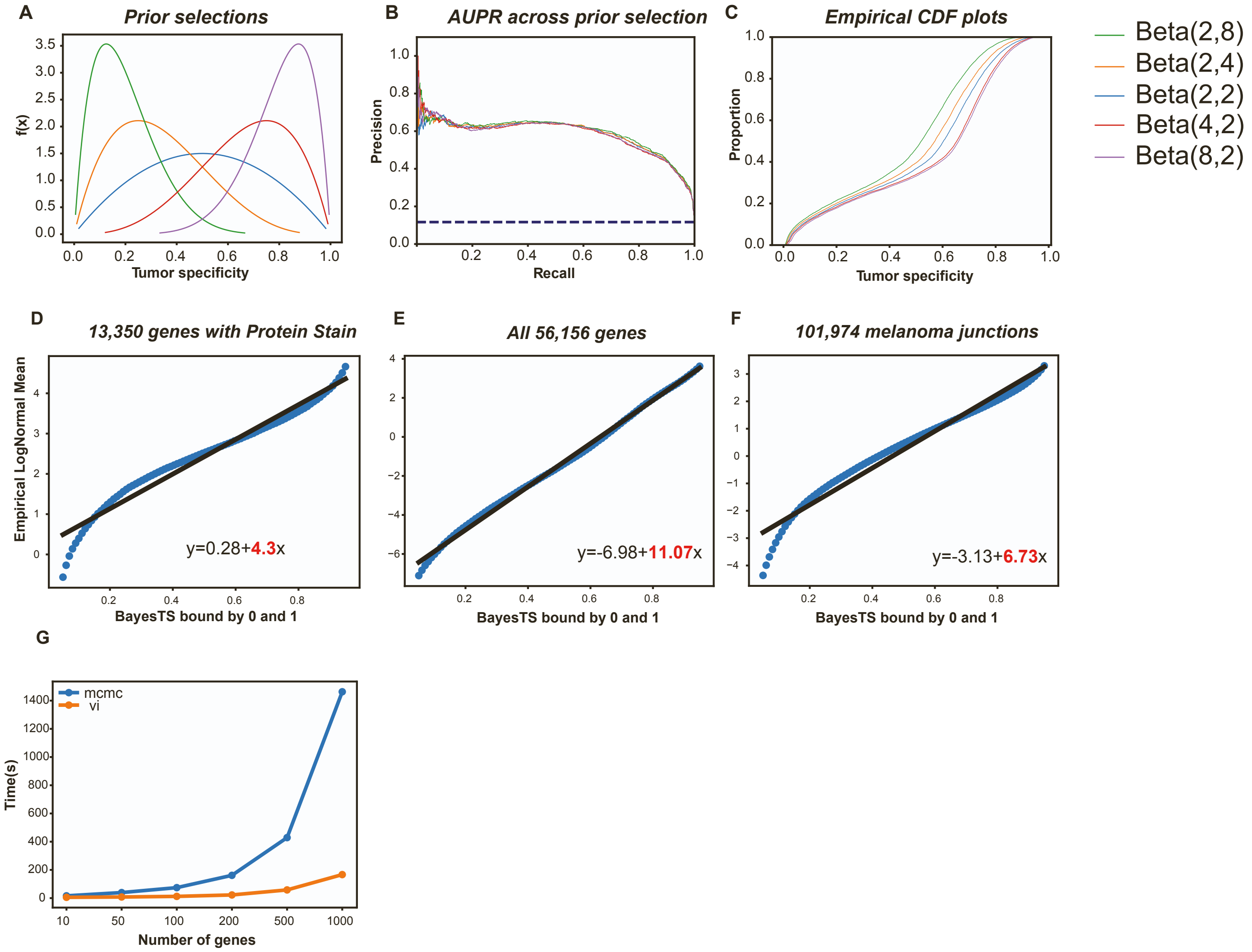

Supplementary Figure 5. Sensitivity analysis, empirical prior determinations and computing performance on different solvers. A) five tested Beta distribution corresponding to decreasing beliefs on tumor-specificity. B) Overall performance on identifying known safe targets across five beta prior. C) eCDF plot of five tested Beta prior on the overall scale of resultant BayesTS score distributions. (D-F) Empirical Bayes approach to derive data-dependent coefficients to account for magnitude difference between sigma and the mean of lognormal distribution to model normalized counts data. (G) Speed comparison on a simplified model and subsampled gene list on pymc5 implemented NUTs MCMC and VI approach. Related to STAR Method and Figure 3.
