# Supplementary material for: Quantifying tumor specificity using Bayesian probabilistic modeling for drug and immunotherapeutic target discovery
Source: Cell Rep Methods. 2024 Nov 7;4(11):100900. doi: 10.1016/j.crmeth.2024.100900 (PMC11705768; doi:10.1016/j.crmeth.2024.100900)
Supplement: Document S2. Article plus supplemental information [file mmc7.pdf]

Cell Reports Methods

Article

Quantifying tumor specificity using Bayesian probabilistic modeling for drug and immunotherapeutic target discovery

Graphical abstract

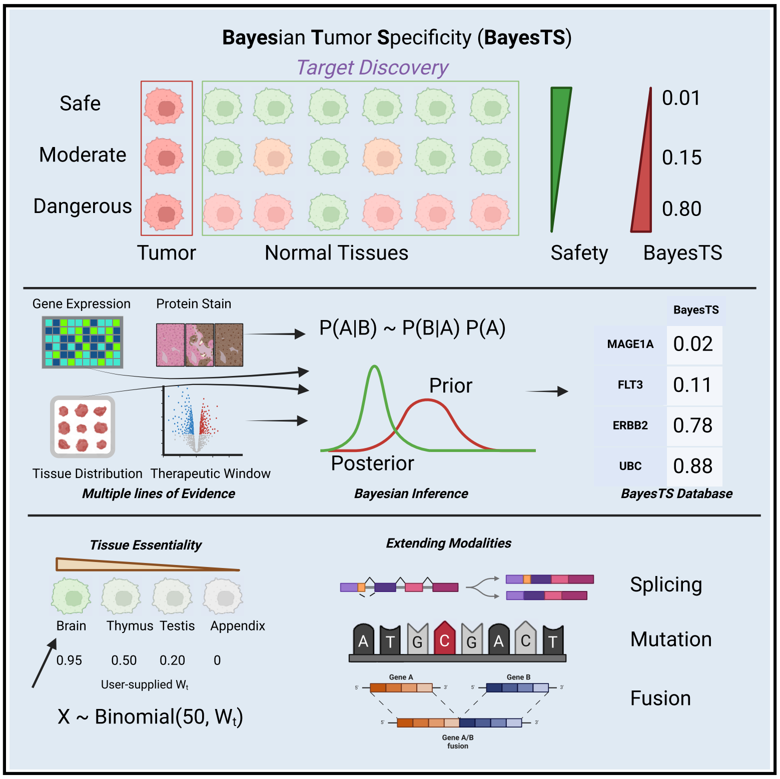

Highlights

- BayesTS is a tunable approach to estimate tumor specificity from omics data
- BayesTS leverages multi-omic evidence, overcoming current limitations
- This approach can be adapted to diverse molecular modalities and questions
- Early embryonic, placental, and germline genes exhibit high tumor specificity

Authors

Guangyuan Li, Daniel Schnell, Anukana Bhattacharjee, Mark Yarmarkovich, Nathan Salomonis

Correspondence

guangyuan.li@nyulangone.org

In brief

Li et al. introduce BayesTS, a Bayesian tumor specificity index that integrates multiple evidence types, including gene expression, protein detection by immunostaining, and tissue distribution, to prioritize targets for immunotherapy. BayesTS offers flexible tuning for safe target selection in cancer that can be extended to nearly any modality.

Li et al., 2024, Cell Reports Methods *4*, 100900
November 18, 2024 © 2024 The Author(s). Published by Elsevier Inc.
https://doi.org/10.1016/j.crmeth.2024.100900

CellPress

Cell Reports Methods

CellPress
OPEN ACCESS

Article

Quantifying tumor specificity using Bayesian probabilistic modeling for drug and immunotherapeutic target discovery

Guangyuan Li,[1,2,3,4,*] Daniel Schnell,[1] Anukana Bhattacharjee,[1] Mark Yarmarkovich,[3] and Nathan Salomonis[1,2]
[1]Division of Biomedical Informatics, Cincinnati Children's Hospital Medical Center, Cincinnati, OH 45229, USA
[2]Department of Biomedical Informatics, College of Medicine, University of Cincinnati, Cincinnati, OH 45267, USA
[3]Perlmutter Cancer Center, New York University Grossman School of Medicine, New York, NY, USA
[4]Lead contact
*Correspondence: guangyuan.li@nyulangone.org
https://doi.org/10.1016/j.crmeth.2024.100900

MOTIVATION Selecting immunotherapeutic targets with tumor-specific expression is crucial for effective immunotherapy and minimized off-target toxicity. Current strategies often rely on arbitrary cutoffs across multiple modalities, introducing subjectivity and hindering cross-study consistency. We present BayesTS, a Bayesian index that flexibly integrates diverse molecular and tissue data while accounting for inferential uncertainty. BayesTS accurately captures expression profiles across modalities and can identify both previously unknown and established targets. Its adaptability extends to unconventional targets, such as splicing events, offering a valuable tool for advancing cancer therapy development.

SUMMARY

In diseases such as cancer, the design of new therapeutic strategies requires extensive, costly, and unfortunately sometimes deadly testing to reveal life threatening off-target effects. We hypothesized that the disease specificity of targets can be systematically learned for all genes by jointly evaluating complementary molecular measurements of healthy tissues using a hierarchical Bayesian modeling approach. Our method, BayesTS, integrates protein and gene expression evidence and includes tunable parameters to moderate tissue essentiality. Applied to all protein coding genes, BayesTS outperforms alternative strategies to define therapeutic targets and nominates previously unknown targets while allowing for incorporation of new types of modalities. To expand target repertoires, we show that extension of BayesTS to splicing antigens and combinatorial target pairs results in more specific targets for therapy. We expect that BayesTS will facilitate improved target prioritization for oncology drug development, ultimately leading to the discovery of more effective and safer treatments.

INTRODUCTION

Tumor cells undergo extensive genetic and genomic alterations that substantially alter their cell growth and metabolism.[1,2] While advantageous for tumor survival, these alterations also introduce potential molecular vulnerabilities that can be exploited for the design of advanced targeted therapies. For example, imatinib, a tyrosine kinase inhibitor, is an effective targeted cancer therapy that acts by selectively binding with the BCR-ABL1 fusion protein in chronic myelogenous leukemia malignant cells, substantially improving clinical outcomes.[3–5] Existing cancer immunotherapies such as checkpoint inhibitors and adoptive cell transfer have significantly increased the repertoire of targets for cancer therapy by leveraging a patient's own immune system as "living drugs."[6,7]

While promising clinical results have been reported in multiple malignancies,[8–11] the efficacy of both targeted and immunotherapy approaches depends on the improved selection of molecular targets, ranging from cancer surface proteins, human leukocyte antigen (HLA)-presented neoantigens, and previously unknown tumor-specific epitopes.[12] Target specificity is frequently best assessed on the basis of target gene or protein expression in diseased versus non-diseased tissues. Indeed, selective evaluation of non-diseased tissues alone can inform tumor-specificity prediction.[13] Importantly, "on-target off-tumor" toxicity can result in severe side effects that can have significant morbidity and mortality when a therapy is evaluated in clinical trials.[13,14] For example, neurotoxicity is a known side effect of chimeric antigen receptor T cell (CAR-T) therapy, with a potential mechanism identified

This is an open access article under the CC BY license (http://creativecommons.org/licenses/by/4.0/).

through single-cell genomics of brain mural cells, which express the CAR-T target CD19.[15]

Quantifying the tumor specificity of drug and immunotherapeutic targets remains challenging due to both significant biological and technological factors. Statistical approaches that estimate tumor specificity are typically binary, requiring predetermined thresholds to estimate the presence or absence of a transcript in healthy reference samples.[13,16] This approach is problematic for multiple reasons. Genes are expressed at different levels and detected at varying levels of sensitivity, depending on the assay employed.[17] Gene expression often does not correlate with protein abundance as a result of cytoplasmic RNA stability, translation potential of the RNA isoform, protein cellular localization, and post-translational regulation.[18] Hence, relying solely on one single molecular modality and quantification approach is limiting when gauging target specificity.

For RNA sequencing experiments, shallow sequencing often underestimates the abundance of transcripts, resulting in false positive tumor-specific predictions. Established best practices to identify tumor-specific targets rely on an array of fixed or tunable thresholds, not considering tissue-specific or cell-type-specific variance in reference to healthy controls.[13,19] An alternative approach is to compute differential expression comparing tumors and healthy control tissues or to define markers most restricted to tumor tissues. These methods include DESeq2, which employs a negative binomial distribution to model observed gene counts and stabilizes variance through a generalized linear model,[20] along with limma and edgeR packages, which leverage empirical Bayes estimations.[21,22] While these methodologies are theoretically valuable for discerning transcripts enriched in tumors, their focus is on identifying tumor-associated antigens rather than tumor-specific antigens. This is because an ideal target should be absent from healthy tissues.

The distribution of gene expression is highly skewed for a large proportion of genes, making a fixed threshold unable to accurately capture the underlying biology.[20] While recent neural network-based methods could be applied to learn and predict tumor specificity, such models are not sufficiently interpretable and do not account for uncertainty to be explicitly quantified. Alternative methods such as bootstrapping offer a valid approach to quantify uncertainty. However, these methods involve trade-offs, including increased computational demands and challenges in defining an appropriate background distribution. Semiparametric models that permit flexible covariate relationships[23] (i.e., splines) and partial specification of variance structure[24] (i.e., generalized estimating equations) have been well studied and widely applied in survival and longitudinal analyses.[25] Bayesian probabilistic approaches offer another framework to address this problem since they are able to incorporate prior knowledge and uncertainty in a structured manner and provide interpretable estimates/predictions and quantification of uncertainty.[26] For example, such modeling in single-cell genomics,[27,28] compositional analysis,[29] and spatial deconvolution[30] has proven valuable, where missing and heterogeneous molecular measurements are inherent. While Bayesian methods effectively circumvent the need for fixed or tunable thresholds, they require careful specification of prior distributions and parameters. This can pose challenges, particularly in small datasets where the influence of the prior may dominate, or when dealing with complex likelihood functions that may lead to inconsistencies. Such issues necessitate a thorough evaluation to ensure the validity of the model's conclusions.

Here, we describe a tunable and quantitative tumor specificity score called Bayesian Tumor Specificity (BayesTS), using probabilistic modeling. Bayesian probabilistic modeling is well suited to quantify target specificity given prior knowledge of the distribution of multimodal measurement data, which can be incorporated into a single coherent model. This approach was designed to handle missing data and to explicitly quantify uncertainty in any supplied molecular measurement data. Despite the potential limitations associated with Bayesian models, we demonstrate that the size of our cohort robustly supports the inference capabilities of the Bayesian framework. Furthermore, our training process effectively integrates diverse lines of evidence, thereby mitigating issues related to non-convergence. BayesTS can successfully learn from multiple molecular modalities and can differentiate between proven-safe versus high-risk CAR-T therapy targets. We show that BayesTS enables accurate dynamic tuning of tissue importance and can identify previously unknown tumor-specific targets. This approach can be generalized further to additional modalities aside from gene products, such as splicing neoantigens, which represent promising targets for therapy. Our proposed BayesTS score is efficient and scalable, using variational inference and leveraging the power of the PyTorch framework to run on graphics processing units[31,32] with effective subsampling. These features enable BayesTS to compute tumor specificity scores for large datasets of 100,000 targets in less than 10 min.

RESULTS

Hierarchical Bayesian modeling to assess tumor specificity

To infer tumor specificity from diverse molecular modalities with distinct forms of relationship to specificity, we developed a hierarchical Bayesian model (BayesTS) without hard-coded thresholds. This model is applied to only non-diseased samples to estimate the probability of a gene/protein being expressed in at least one tissue type. Molecular evidence includes RNA genomic quantification (RNA sequencing [RNA-seq]) and protein abundance (immunohistochemistry) (STAR Methods). The distribution of samples in the reference dataset of normal controls is assessed at a population and tissue level, accounting for varying numbers of replicate controls (Figure 1). The model was developed to allow for flexible weighting of tissue essentiality in the model, which might be considered as less or not important from the perspective of therapeutic targeting (e.g., testis-specific expression in female-coded samples). Since BayesTS inferred sigma represents the level of presence in normal tissue, unless specifically noted in the paper, a lower BayesTS score indicates high tumor specificity.

BayesTS accurately estimates tumor specificity from multidomain evidence

We trained the BayesTS model using three observations: (1) a normalized RNA count matrix, (2) tissue distribution profiles,

CellPress
OPEN ACCESS

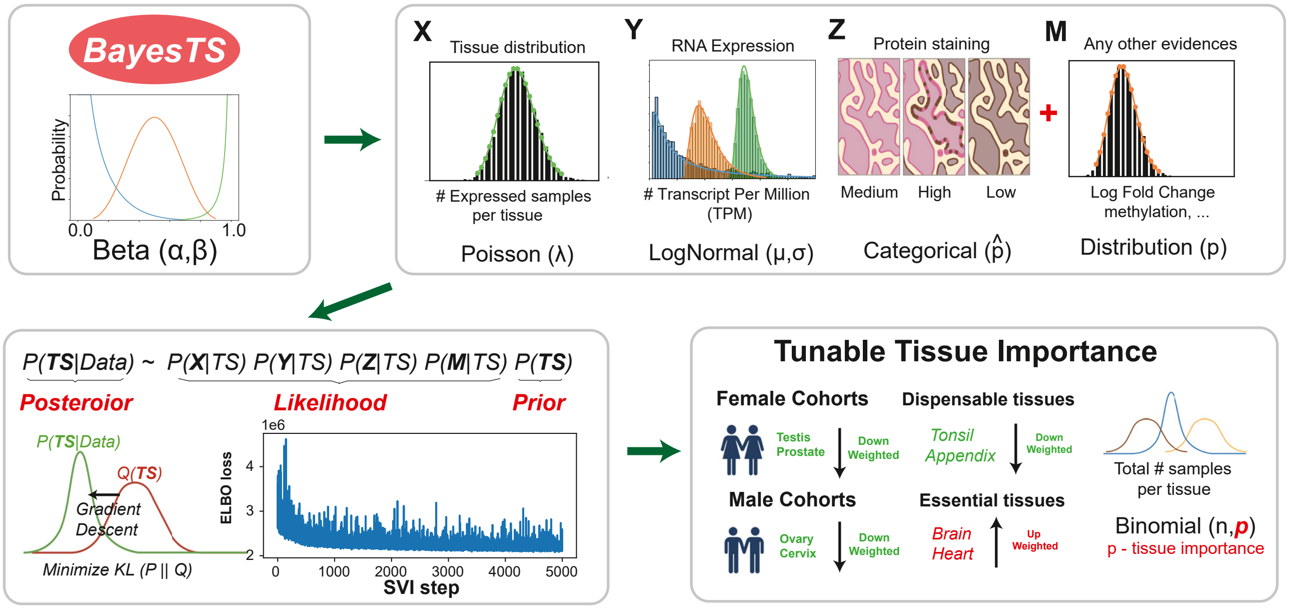

Figure 1. BayesTS automates the identification of tumor-specific proteins from multiple data dimensions

A single continuous value (BayesTS) bounded by 0 and 1 gives rise to three domains of evidence, including the number of samples expressing a specific target per tissue (X), the normalized RNA count values across multiple samples (Y), and immunohistochemistry evidence indicating whether a protein is confidently detected (Z), along with user-supplied additional modalities (M). Variational inference was used to effectively approximate the true posterior through gradient descent. Tunable user-supplied tissue importance options enable flexible modeling of tumor specificity.

and (3) protein expression labels (STAR Methods). We ensure that each independent piece of evidence is considered equally in the inference process by scaling their log probability based on the maximum loss when training them alone (STAR Methods). The BayesTS model converges well after around 2,000 steps, with gradually decreasing evidence lower bound (ELBO) loss, indicating that the model successfully updated the weights toward the observed data (Figure S1A). To assess whether it can leverage all three pieces of evidence, we project the inferred posterior tumor specificity onto a three-dimensional space where each line of evidence represents a separate axis. BayesTS is capable of capturing evidence gradients along all three axes to nominate potential targets (Figure S1B). To demonstrate the importance of considering multiple types of tumor-specificity representations, we performed a leave-one-out sensitivity analysis by removing each modality, one at a time, to evaluate the impact on the final inferred scores across all genes or focusing on 54 CAR-T clinical targets. As expected, when protein information was left out of the model, no gradient can be seen along the z axis, with similar observations along other axes when normalized counts or tissue distributions are not provided to the BayesTS model (Figure S1C; Table S1).

To benchmark the performance of BayesTS, we set out to curate a list of high-confidence tumor-specific genes by systematically reviewing known sources, including CAR-T targets, cancer testis antigens (CTAs), and lowly expressed genes, which resulted in 3,845 evidenced "safe" targets (Figure 2A). Since Bayesian modeling using variational inference is stochastic, we first confirm the reproducibility and stability of the inferred BayesTS scores by independently running the model five times. This analysis confirmed that BayesTS scores are indeed stable, with negligible differences observed across our trials (Figure 2B). To assess the utility of considering multiple molecular modalities, we tested each modality separately and together. This analysis showed that multimodal analysis results in the best BayesTS performance for prioritizing evidenced "safe" targets (area under the precision-recall curve [AUPR] = 0.59), as compared to only considering tissue distribution (AUPR = 0.54), RNA expression evidence alone (AUPR = 0.43), or protein staining evidence alone (AUPR = 0.43) (Figure 2C). While we are not aware of a prior dedicated tumor-specificity scoring approach, to further assess the value of Bayesian modeling for this problem, we adapted a previously noted tau score, which can be used to distinguish whether a gene is tissue specific or is ubiquitously expressed in multiple tissues where the tissue-specific genes are deemed more "safe" compared to targets expressed in profiled tissues.[33] Comparing the Human Protein Atlas (HPA) reported tau-based tissue specificity scores to BayesTS, BayesTS again showed superior performance (Figure 2D).

To examine the extent to which the proposed BayesTS accurately reflects the tumor specificity of known drug targets we curated 54 CAR-T therapy drug targets that are currently under at least one National Cancer Institute (NCI)-registered clinical trial (STAR Methods). In this set, we find that BayesTS ranked the well-established CTAs including MAGEA1 and MAGEA4 as the most tumor-specific targets. Since testis is the only organ in healthy adult tissues expressing CTAs, they have been used in the treatment of multiple cancers,[34,35] followed by the highly tumor-specific antigens ALPP[36] and CLDN18[37] (Figure S1B). We note that not all CAR-T targets have a highly

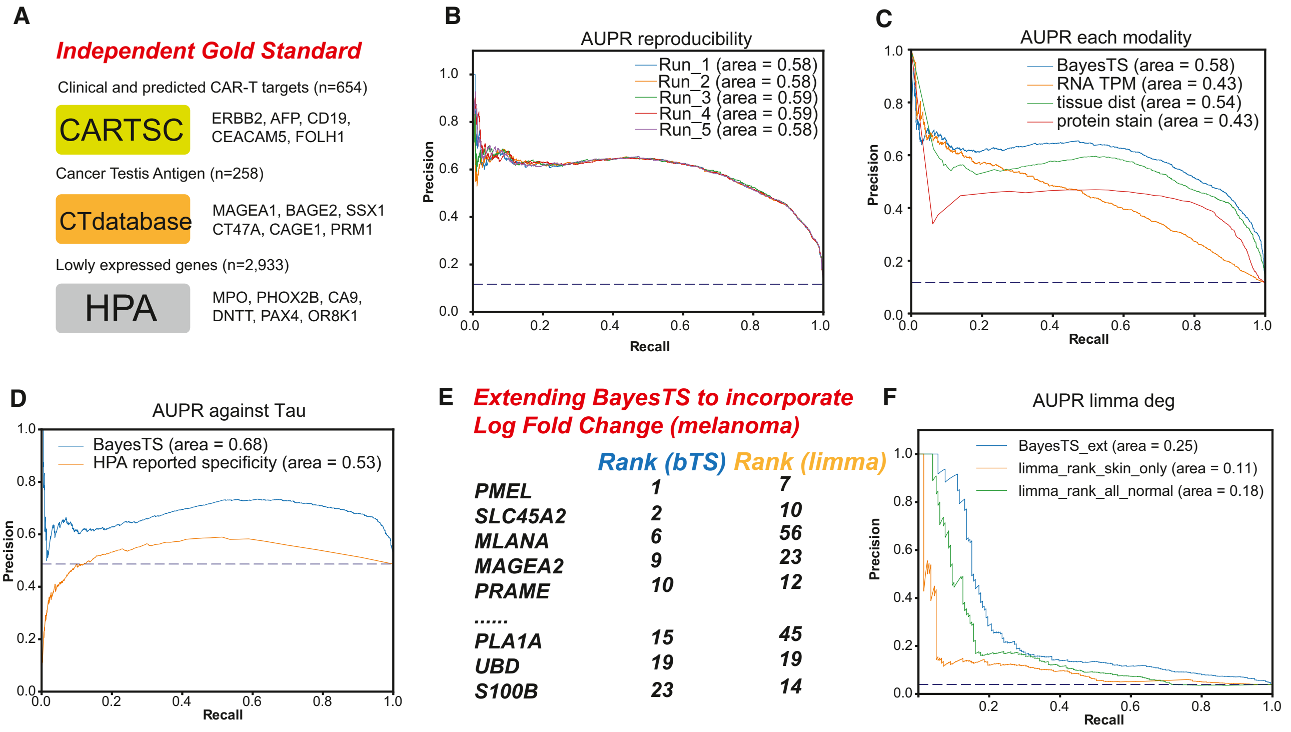

Figure 2. Reproducibility and evaluation of BayesTS

(A) Curation of best-evidence "safe" targets for targeted cancer therapy.

(B) Area under the precision-recall curve (AUPR) of five independent runs of BayesTS.

(C) AUPR of BayesTS by combining all three lines of evidence compared to using each modality alone.

(D) AUPR of BayesTS against previously described HPA-reported tissue specificity tau scores.

(E) Extending BayesTS to directly incorporate tumor gene differential expression (melanoma versus healthy skin or all tissues, log fold change), using the shown prior defined tumor-specific antigens (HPA). BayesTS (bTS) is compared to simple limma differential expression analysis, by ranked order comparison.

(F) AUPR of the extended BayesTS model and limma ranks in prioritizing known melanoma-specific antigens.

restricted tumor expression profile. Instead, complex optimization steps, such as affinity optimization and drug influx and efflux considerations, are typically used to ensure that the developed therapy targets only tumor cells.[38,39] A recently reported peptide-centric CAR strategy targets the intracellular oncogenes PHOX2B that can generate a self-antigen (QYNPIRTTF) presented on multiple HLA-I alleles, rendering it a promising cancer target in a wide range of neuroblastoma patients.[40] PHOX2B, as a validated neuroblastoma-specific gene, was assigned an extremely low BayesTS score (0.03), consistent with the preclinical safety testing of this therapy and supporting the capacity of BayesTS to prioritize valid cancer targets. Nevertheless, drug-targeting proteins with widespread expression in normal tissues have been reported to cause severe on-target off-tumor toxicity.[41] Consistent with our sensitivity analysis, when trained using incomplete evidence, the modality excluded is rendered less essential in inferred tumor specificity (Figure S1B; Table S2). Importantly, we observe partial correlations between tissue distribution and normalized RNA counts such that even if one piece of evidence is excluded, the overall pattern remains. However, the protein-level information is more independent and exhibits a nearly random distribution when it is not included in the BayesTS model (Figure S1C). This again highlights the need to incorporate multiple pieces of evidence rather than relying on one.

An important validation for any Bayesian model is to inspect whether the model learns from the data and updates the prior belief to better reflect the properties of the observations.[42] To confirm that the model learns properly, we conducted a prior and posterior check by first generating samples from the underlying prior and posterior distribution of the tumor specificity, and compared these against the actual data distributions. As a lowly expressed target in normal tissue and a well-characterized CTAs in solid tumors,[43] the tumor specificity of MAGEA1 shifts from 0.49 to 0.02 and reflects a clear left-skewed distribution for both the tissue distributions and normalized RNA counts (Figure S2A). When evaluated on the protein-level data, the sampled labels from the posterior have a higher proportion on the "low" label (numerically labeled as 3) compared to the prior distribution, demonstrating that the model appropriately incorporates information from the data (Figure S2A). In contrast, a highly expressed target, UBC, shifts its tumor specificity from prior 0.50 to 0.86 due to the high counts for both its RNA and protein (Figure S2B). Here, we show that BayesTS successfully learns from multiple lines of evidence and reflects the true distributions for established drug targets.

Integration of tumor gene expression

As the default BayesTS database does not explicitly consider target gene expression in disease samples, but rather only in healthy controls, we assessed its performance gain by including tumor versus matched control log fold change (LFC) in the model. LFC values were obtained from the Gene Expression Profiling Interactive Analysis (GEPIA) web portal, which enables direct comparisons between Genotype-Tissue Expression (GTEx) and The Cancer Genome Atlas (TCGA) tumor samples that have been uniformly processed.[44] We compared the ability of the extended BayesTS model with limma-based differential expression (p value and effective size) in melanoma versus healthy skin (not considering other healthy tissues), to assess which method best identifies previously established melanoma-specific target genes. Here, we defined melanoma-specific antigens annotated in the HPA (STAR Methods). As expected, BayesTS with this extended LFC model has better performance (AUPR = 0.25) than the limma-generated ranks (AUPR = 0.11; Figures 2E and 2F). We then further included all normal tissues as the melanoma reference in addition to healthy skin. While the limma-derived AUPR increases when using all tissues, it is still outperformed by BayesTS, further suggesting the importance of explicitly modeling the normal tissue expression. The relatively lower AUPR is likely due to limited curation of melanoma-specific genes in HPA. For instance, CSAG3 is the only falsely prioritized antigen in the top 20 list of BayesTS prediction. However, despite its absence in normal tissue and upregulation in melanoma, it is not annotated as a known melanoma-specific antigen. Thus, BayesTS improves the definition of tumor-specific genes considering both the presence and absence of tumor gene expression profiles.

Considering tissue essentiality to define expanded drug targets

As our first application, we aimed to evaluate the ability of BayesTS to integrate information related to tissue essentiality. For cancer therapy, some tissues are physiologically irrelevant, as expressed proteins will not be immunogenic or can be readily renewed. For example, sex-specific tissues can be selectively down- or upweighted to accurately reflect the enrolled patient's tissue type. Additionally, physiologically expendable tissues such as tonsils and appendices, or organs that are typically excised in certain cancers, such as the adrenal gland in neuroblastoma,[45] can be preferentially downweighted in specific scenarios. Other clinically relevant examples include CDH17, a known CAR-T target that has been shown to be safe despite its expression in the gastrointestinal (GI) tract.[46] This safety is due to the tight junctions in normal tissue, which make CDH17 less accessible. Another example is ocular immune privilege, where the immunosuppressive environment of the eye reduces the likelihood of CAR-T cells reacting.[47] BayesTS empowers users to incorporate prior knowledge of tissue importance to address real-world use cases effectively. Using a well-known cancer testis antigen, PRAME, as an example, when upweighting (weight = 0.9) and downweighting (weight = 0.1) the testis tissue importance, the inferred BayesTS score consistently reflected the changes ($p = 4.04e-7$; paired t test), without violating the overall conclusion that it is still a valid drug target (Figure 3A). Next, the tumor specificity for the widely recognized hematological malignancy CAR-T target,[48,49] CD19, also significantly decreases when selectively downweighting the immune cell-producing blood cells and spleen ($p = 8.54e-6$; paired t test). This approach is particularly relevant because it has been demonstrated to generate a relatively safe clinical response regardless of the presence of the remaining immune populations.[13] Lastly, we evaluate the ability of BayesTS to reflect the CDH17 use case when adjusting for GI tract weights. As expected, the CDH17 BayesTS score significantly decreases when the GI tract weights are reduced from 0.9 to 0.1 ($p = 3.82e-5$; paired t test). While the score decrease is relatively minor, this demonstrates the utility of this approach (Figure 3A). The flexibility of BayesTS makes it easily extendable in complex clinical trial designs and drug prioritizations.

As the primary application for a multifaceted tumor specificity score, BayesTS enables rapid discovery and prioritization of known and previously unknown drug targets. When ranking the tumor specificity of 13,350 genes with protein information in ascending order, targets falling within similar tumor specificity (BayesTS = 0.02) as the well-known targets (e.g., MAGEA1, MS4A1) can be selected for further evaluation (Figure 3B). To identify previously unknown targets, we analyzed TCGA skin cutaneous melanoma (SKCM) with 472 patients in total and compared the gene expression differences of nominated drug targets based on inferred tumor specificity score. First, we found multiple MAGE family proteins (e.g., MAGEC2, MAGEB2, MAGEC1, MAGEB6) with a gene expression profile similar to those in clinical trials (MAGEA1, MAGEA4), which have been reported to be tumor specific.[50–52] Second, we found numerous pregnancy-specific glycoprotein genes, which fall into the broader category of carcinoembryonic antigens (Figure S2C). While the association of placenta trophoblasts and tumor have been suggested previously,[53] these targets have not been included in active CAR-T clinical trials; hence, they may represent additional unexploited targets for melanoma treatment. Aside from these previously reported potential antigens, we nominated a histone methyltransferases gene (PRDM7) that is restricted in its expression to testis, but is otherwise uniquely expressed in melanoma tumors. PRDM7 is responsible for regulating DNA methylation and accessibility (H3K4me2 to H3K4me3), and hence could play a role in tumor homeostasis[54] (Figure 3B). In addition, these analyses highlight a number of previously unknown targets that are expressed in melanoma (RNA-seq) but have evidence of a lack of expression in healthy tissues from GTEx, TCGA controls, and HPA. These include the N-terminal acetyltransferases (NAA11), a suppressor of circadian rhythm (PASD1), and the suppressor of microRNA biogenesis (LIN28) (Figure S2C). Hence, such proteins represent intriguing high-value targets for validation in independent control tissue datasets.

Finally, to demonstrate that BayesTS can be readily applied to distinct molecular targets, we applied this model to a large dataset of tumor-detected alternative splicing events (melanoma). Tumor-specific splicing events have been implicated in oncogenic transformation and metastasis[55] and are often associated with clinical outcomes.[56] Such targets include splicing neoantigens, which are highly specific exon-exon or exon-intron junctions that encode for immunogenic major histocompatibility

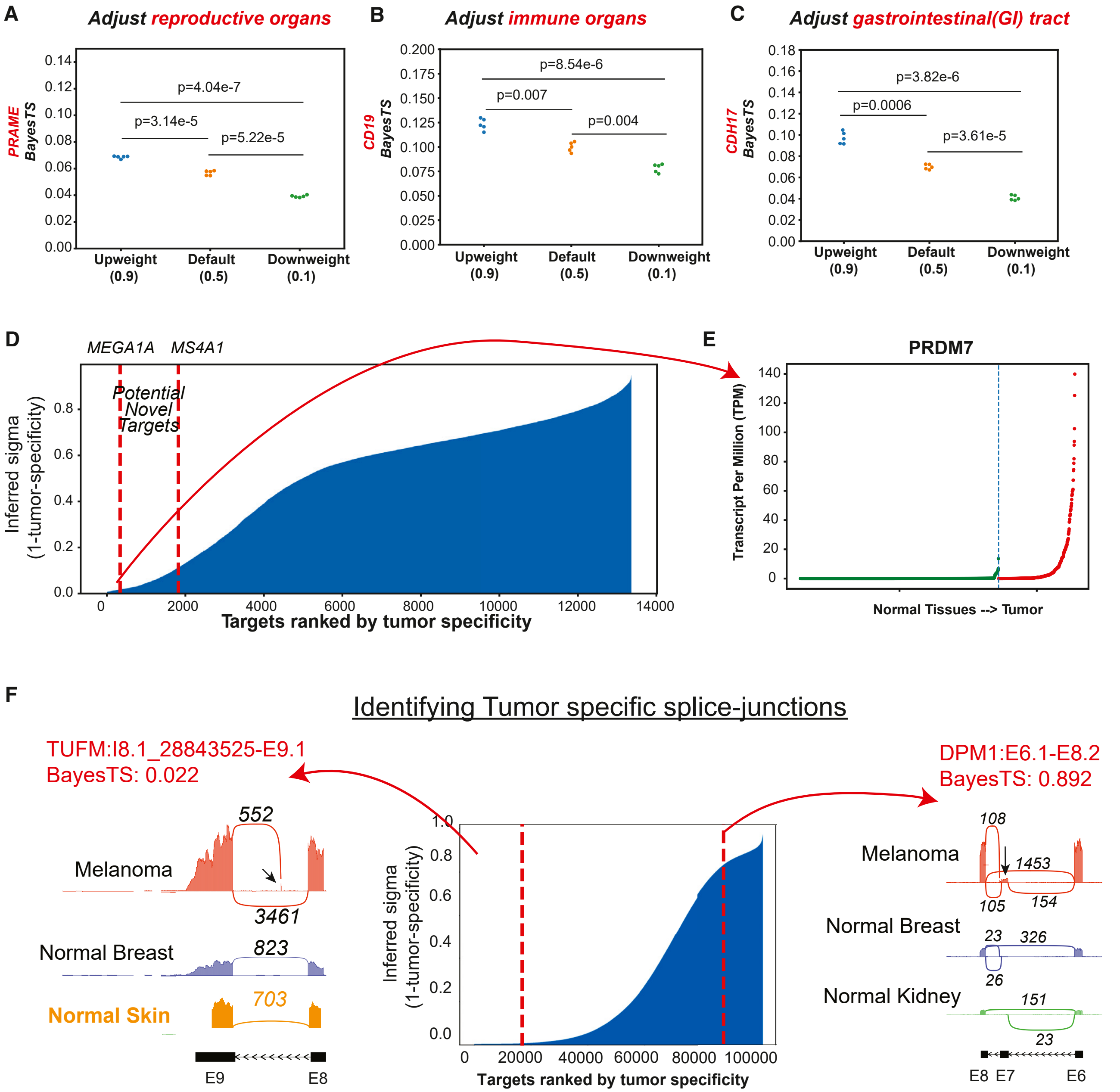

Figure 3. Adjusting tissue importance in the model to identify previously unknown gene targets and unconventional drug targets

(A–C) Adjusting tissue importance for selected tissues. Here, we display the inferred changes in sigma (y axis) after adjusting the tissue importance of (A) testis, (B) immune organs, and (C) gastrointestinal tract. *p* value was derived using paired t test and annotated in each panel.

(D) Identification of previously unknown drug targets in melanoma from the inferred tumor specificity scores by ranking all targets in ascending order.

(E) Melanoma-specific expression for PRDM7.

(F) Identification and comparisons of unconventional splice junctions. A tumor-specific previously undocumented exon event (left, not in normal skin as well) and an exon-skipping event that are present in normal tissues (right).

complex-presented peptides, representing additional targets for cancer vaccine development.[12,57] We analyzed 472 TCGA SKCM patients and quantified individual sample splice junction expression. Similar to our gene-focused pipeline, we trained the BayesTS model using only tissue distributions and normalized RNA counts for GTEx and TCGA control splice junctions. Here, we were not able to supply associated protein quantifications for these junctions, which require highly sensitive and specific junction-level mass spectrometry profiles. We re-derived the scaling factors by training each line of evidence (Figure S3A). The scaling factors ensure that each line of evidence contributes equally to the inference. When combined, BayesTS learns

CellPress
OPEN ACCESS

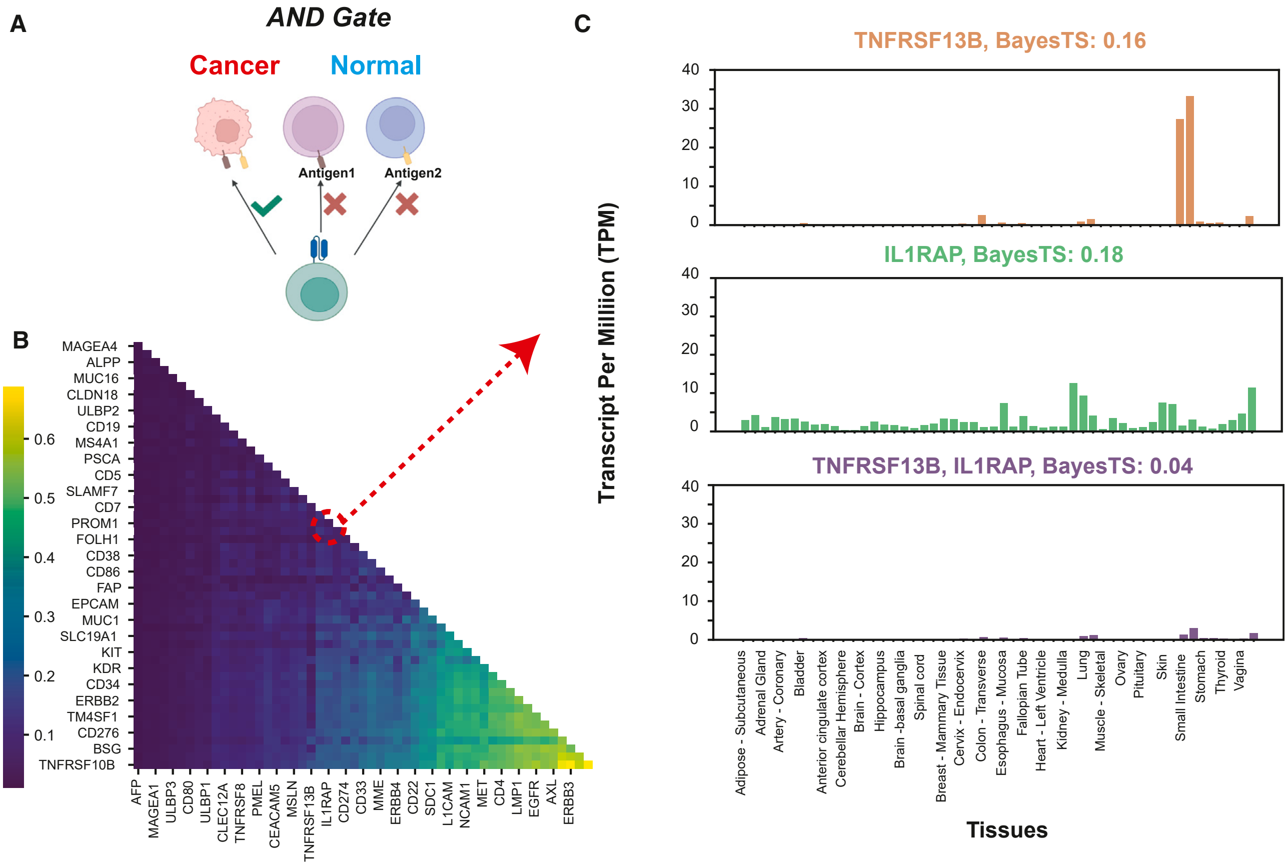

Figure 4. BayesTS identifies suitable target pairs for logic gate CAR-T therapy

(A) Schematic overview of AND gate target pair identification. Here, the specificity refers to the simultaneous presence of two targets.

(B) Heatmap of BayesTS score for all 1,431 possible target pairs for 54 known CAR-T targets (in at least one National Cancer Institute clinical trial).

(C) Example tissue expression profiles for TNFRSF13B and IL1RAP, together with their combined tumor specificity and BayesTS score.

toward the observed data, while leaving one modality out fell short of capturing the corresponding information (Figures S3A and S3B). We highlight two splice junctions with opposite tumor specificity verified by SashimiPlot visualization. These include a previously undocumented exon of gene TUFM (AltAnalyze: I8.1_28843525-E9.1) that is highly specific to melanoma patients (BayesTS score = 0.029), whereas an exon-skipping event DPM1 (E6.1–E8.2) is broadly present in healthy tissues (BayesTS score = 0.864) (Figures 3C; Table S3). Hence, BayesTS is broadly applicable to different molecular modalities and molecular specificity contexts.

BayesTS identifies combinatorial targets for logic AND gate

Although the identification of a single target for effective cancer immunotherapy is ideal, it remains challenging to find candidates with desirable cancer dependencies that minimize immune evasion and mitigate the risk of on-target off-tumor effects.[58] A recent pan-cancer analysis suggested that the current CAR-T single-arm therapy approaches have reached near saturation.[59] In light of this, combinatorial target design has been explored and validated as a means to implement safer immunotherapies and reduce the likelihood of cancer relapse.[60] The logic-gated strategy is a commonly used approach in CAR-T therapy design, where the goal is to identify safe target pairs that are co-expressed in tumors that are not present in healthy tissues[61] (Figure 4A). Various engineering techniques, such as synNotch,[62] splitCAR,[63] and LINK CAR,[64] have made this possible. In particular, LINK CAR employs a strategy by splitting the intracellular proximal T cell signaling molecules SLP-76 and LAT, with each protein associated with the receptor for one target, in lieu of the traditional CD3 ζ chain to trigger the downstream T cell activation. Despite the potential of increasing the therapeutic window through targeting antigen pairs in tumor cells, an ongoing challenge is the effective identification of optimal target pairs. In this context, BayesTS can streamline the process by considering multiple pieces of evidence, thereby eliminating the need to define different cutoffs to enforce tumor specificity. This approach reduces subjectivity and allows for more standardized and comprehensive benchmarking.

To demonstrate the ability of BayesTS to identify safe combinatorial targets, we selected 54 CAR-T targets that have been involved in at least one NCI-registered clinical trial, suggesting their individual suitability.[19] We hypothesize that the combination of these 54 CAR-T targets would produce improved tumor specificity and identify safer targets for cancer immunotherapy. When both

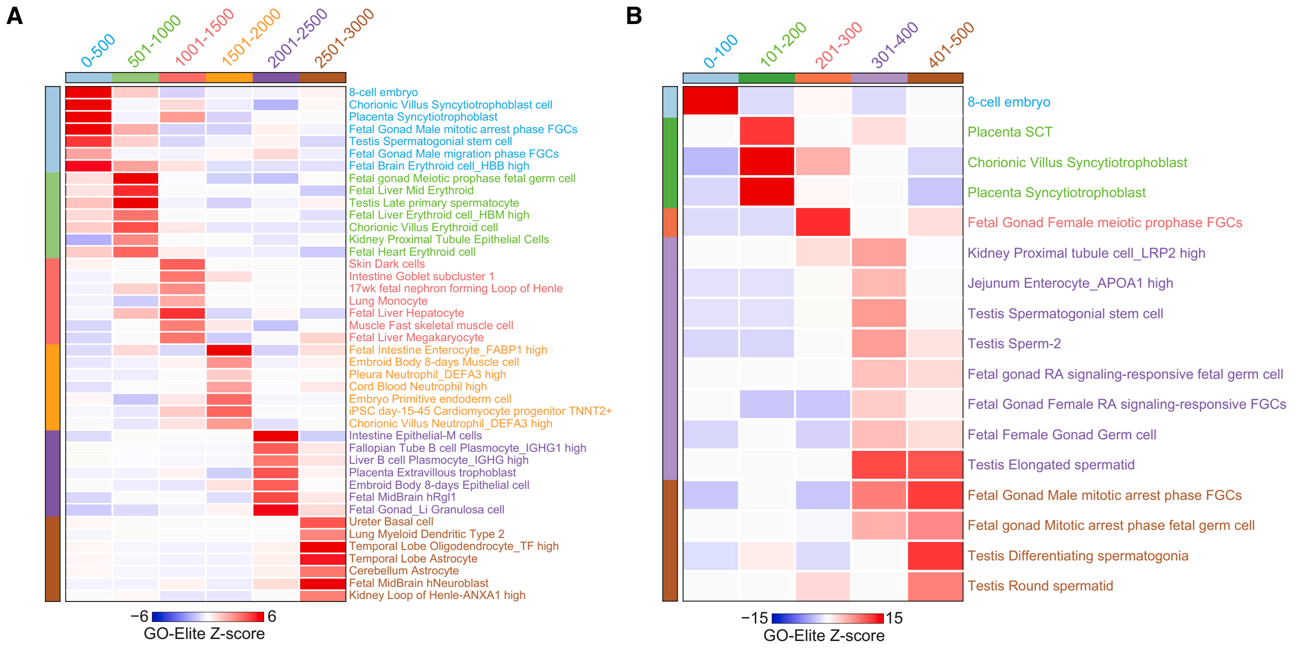

Figure 5. Tumor-specific genes are uniquely expressed in early embryonic and developmental cell types

Heatmap of GO-Elite gene set enrichment of cell state-specific biomarkers for the top-ranked BayesTS tumor-specific gene sets (highest to lowest, left to right) for (A) 500 gene bins and (B) 100 gene bins.

targets are expressed in a specific sample, we choose the lowest expression value as a surrogate for that target pair. This procedure was applied to mRNA gene expression, tissue distribution, and protein levels. BayesTS inference applied to the target pairs identified 797 pairs with a BayesTS score of <0.1, indicating AND gate specificity, with only 10 single CAR-T targets matching this criterion (Table S1). Target pairs predicted to independently encode for highly tumor-specific antigens (e.g., MAGEA1, MAGEA4, CLDN18) unsurprisingly resulted in highly tumor-specific antigen pairs as well (Figure 4B). However, we discovered 671 target pairs with a significant reduction in BayesTS scores ($\delta > 0.2$) compared to single antigens (Table S1). Notably, TNFRSF13B and IL1RAP, when examined separately, yielded a BayesTS score of around 0.2, with evident high expression in certain tissues. TNFRSF13B was found to be highly expressed in the small intestine, spleen, and pancreas, posing a risk for TNFRSF13B-based therapy, whereas IL1RAP exhibited specific expression in the liver, lung, and esophagus. When combined, the mutual exclusivity of these two targets resulted in a much lower expression profile in normal tissues, with modest presence in the skin, spleen, and thymus (BayesTS = 0.04; Figure 4C). Consequently, employing TNFRSF13B plus IL1RAP could enhance the therapeutic index through logic-gated CAR-T therapy.[65]

Distinct gene signatures associated with different tiers of tumor specificity

As shown, the continuous nature of BayesTS scores enables quantitative and logic-based analyses of tumor specificity relative to arbitrary cutoffs. A compelling question that arises from these predictions is why only certain genes exhibit higher tumor specificity compared to others. While these data could point to a common gene regulatory mechanism, such as global deregulation of DNA methylation, unique inflammatory interactions, or genomic instability, we postulated that tumor-specific genes have developmentally restricted expression, required for cell plasticity and immune evasion. To determine whether genes with similar BayesTS probabilities encode for factors with conserved and coherent genetic, molecular, or regulatory biology, we ranked genes based on their BayesTS score for gene set enrichment analysis. We performed gene set enrichment (GO-Elite) of discrete lists of the top 3,000 BayesTS tumor-specific genes, divided into 500 gene bins, considering a large database of human cell-type-specific signatures from single-cell RNA-seq. The most significantly enriched (Z score) terms were observed in the most tumor-specific genes, associated with embryonic blastocyst (cleavage stage), placental syncytiotrophoblast, and mitotically arrested fetal gonadal germ cells, consisting of over 50 genes, collectively (Figure 5A). In contrast, genes with lower BayesTS scores (sixth-ranked bin), were associated with adult or fetal neuronal, epithelial, and immune programs, suggestive of lineage-restricted programs. Discretizing the top-ranked bin further, we found that the top 100 BayesTS genes are uniquely and specifically associated with the earliest stages of early embryogenesis (Fisher's exact p value $5e-14$, false discovery rate corrected), consisting entirely of 10 cancer/testis antigen family member genes (Figure 5B). Extension of this analysis to other diverse types of gene sets and pathways highlights genes associated with vision and eye disease (e.g., crystallin proteins, photoreceptors and transcription factors), antimicrobial peptides expressed in neutrophils and sperm cells (defense response), spermatogenesis, cytokine signaling and regulation by POU2F1, and nuclear factor κB (NF-κB)

signaling, which are frequently associated with tumorigenesis. For example, crystallin proteins have been shown to inhibit apoptosis and enhance cell survival, while antimicrobial gene expression can contribute to tumor-promoting microenvironments. Similarly cytokine and NF-κB signaling can promote a pro-inflammatory environment conducive to cancer development that leads to enhanced cell proliferation, survival, and metastasis (Table S4). The upregulation of these pathways is likely to positively contribute to tumorigenesis and tumor relapse, as they may create favorable conditions for cancer cell proliferation, invasion, and immune evasion.

DISCUSSION

Accurately predicting tumor specificity is a crucial step in the design of effective therapeutic strategies for diseases such as cancer to improve target prioritization and preclinical evaluation. Here, we describe a principal tumor specificity index, BayesTS, to simultaneously evaluate multiple representations of tumor specificity including both RNA and protein evidence. Using a large dataset of 55 normal tissues spanning over 3,500 samples, we showed that BayesTS can prioritize well-established drug targets, nominate combinatorial target pairs, and illuminate the potential biological mechanisms associated with the tumor specificity profile. BayesTS employs a formal definition of tumor specificity, thereby enabling comprehensive downstream correlation analysis, including discovery of biologically coherent gene modules that denote core cancer dependencies. As an example, we interrogated the relationship between gene tumor specificity and their propensity to harbor diverse genomic variants (substitutions, copy-number variation) or differential methylation. We found a weak but significant correlation between tumor specificity and the splice variants (Pearson $r = 0.21$, $p = 1.75e{-7}$; Table S4; STAR Methods). When clinical trial data are available, BayesTS can be utilized to analyze the correlation between drug target specificity and the success rate. These highlight both the theoretical and practical advantages over existing approaches, which rely on *ad hoc* thresholds and manual inspection. Furthermore, BayesTS can be easily extended to other modalities such as epigenetic[66] and post-translational modification profiles.[67]

While numerous methods exist to identify genes overexpressed in specific cell types or tumor tissues, the development of effective and safe immunotherapies also requires ensuring minimal or no expression in normal tissues. Examples of naive approaches to address this challenge include using log(TPM) (transcripts per million) < 4 to define tumor-specific expression,[13] whereas the HPA uses nTPM (number of transcripts detected for a given gene) <1 as non-expressed,[68] or user-defined, cutoffs for various properties.[16] These methods are often subjective and restricted to a specific study, hindering cross-study comparisons. We generated BayesTS scores for all human protein-coding genes as a reusable resource for the cancer research community. To ensure a fair comparison of the performance of BayesTS, we performed three key benchmarks. First, we compared the ability of BayesTS to identify safe targets by first curating a set of independently reported known cancer therapy targets with low expression in healthy tissues. Second, we confirmed that BayesTS vastly improved multimodal accuracy over single-modality analyses. Third, we demonstrated that BayesTS outperforms independent protein-based specificity metrics (tau) [33]and conventional differential expression approaches (limma[22]), considering joint tumor and healthy samples. We note that unlike tau or limma, which are not designed explicitly to define a lack of tissue-specific expression, these approaches do not consider multiple modalities that improve the predictive power of BayesTS. While demonstrated for protein, genes, and alternative splicing, BayesTS allows for the creation of additional reference datasets using independent modalities, by redefining the input to handle logic-gated target pairs and alternative feature types or serving as a prior for subsequent inferential processes. Moving forward, we anticipate the incorporation of BayesTS and derivative methods into advanced tumor specificity workflows, as recently demonstrated for splicing neoantigen prediction.[69]

Limitations of the study

We note that there are potential limitations to our approach. Although BayesTS provides a parameter-free framework, it is still important to define a reliable prior, given that the resultant score scale can change slightly, depending on the range of prior chosen. It is not always trivial to select the right variational function to approximate the true posterior that can alleviate the bias and reflect the true hierarchical relationship. In terms of our overall assumptions and potential prioritization of tissue-specific genes, ideal tumor targets should represent core tumorigenesis dependencies present in the majority of cancer clones (heterogeneity). Thus, future inclusion of large-scale single-cell permutation results (e.g., PerturbSeq) are likely to further increase the specificity of BayesTS predictions. A second caveat is that our approach assumes that the same detection biases exist in the control and disease tissues evaluated. If significant differences in the RNA or protein quantification exist between the control and disease datasets (e.g., batch effects, library generation, sequencing depth), then this could result in both false positive and false negative predictions. Finally, as our approach has been applied to bulk RNA-seq and protein quantification data, it is likely to be blind to extremely rare biologically crucial cell-type gene expression programs in healthy cells, which express otherwise predicted tumor-specific molecules. This challenge can be overcome through the extension of this approach to large single-cell and disease cohorts with different sample and cell-population biases that can be accounted for automatically in the underlying model. Furthermore, multi-view deep neural network-based representation learning may be capable of capturing complex interactions among different forms of evidence better than explicit Bayesian modeling.[70] However, we expect our Bayesian strategy to be applicable to diverse disease specificity challenges, exploiting both tissue- and cell-level candidate target abundance.

RESOURCE AVAILABILITY

Lead contact

Further information and requests for resources and reagents should be directed to and will be fulfilled by the lead contact, Dr. Guangyuan Li (guangyuan.li@nyulangone.org).

Materials availability

This study did not generate new unique reagents.

Data and code availability

- All the data required to reproduce the study, including the curated gold standard benchmark database, have been deposited to Synapse (https://www.synapse.org/Synapse:syn61670083). The BayesTS database is available at https://github.com/frankligy/BayesTS/tree/main/database.
- BayesTS software is available at https://github.com/frankligy/BayesTS. The source code has been deposited at Zenodo (https://doi.org/10.5281/zenodo.13922316) and is publicly available as of the date of publication. DOIs are listed in the key resources table.
- Any additional information required to reanalyze the data reported in this paper is available from the lead contact upon request.

ACKNOWLEDGMENTS

This work was supported by generous funding from the Cincinnati Children's Hospital Research Foundation and the National Institutes of Health (R01CA226802, to N.S.).

AUTHOR CONTRIBUTIONS

G.L. conceived the method, wrote the software, and performed all bioinformatics analyses. D.S. provided statistical advice. A.B. assisted with data acquisition and processing and quality control analyses. M.Y. advised on the BayesTS application on combinatorial target pairs discovery. G.L., N.S., D.S., and M.Y. wrote the manuscript.

DECLARATION OF INTERESTS

The authors declare no competing interests.

STAR★METHODS

Detailed methods are provided in the online version of this paper and include the following:

- KEY RESOURCES TABLE
- METHOD DETAILS
  - Datasets used in the work
  - BayesTS model
  - Extending BayesTS to custom modalities
  - Deriving a posterior distribution using variational inference
- QUANTIFICATION AND STATISTICAL ANALYSIS
  - Benchmark BayesTS performance
  - Bulk RNA-seq preprocessing
  - Immunohistochemistry preprocessing
  - Prior and posterior check
  - CAR-T therapy targets evaluation
  - Gene enrichment and network analysis

SUPPLEMENTAL INFORMATION

Supplemental information can be found online at https://doi.org/10.1016/j.crmeth.2024.100900.

Received: February 26, 2024
Revised: July 16, 2024
Accepted: October 17, 2024
Published: November 7, 2024

REFERENCES

1. Hanahan, D., and Weinberg, R.A. (2000). The hallmarks of cancer. Cell *100*, 57–70.
2. Hodis, E., Triglia, E.T., Kwon, J.Y.H., Biancalani, T., Zakka, L.R., Parkar, S., Hütter, J.-C., Buffoni, L., Delorey, T.M., Phillips, D., et al. (2022). Step-wise-edited, human melanoma models reveal mutations' effect on tumor and microenvironment. Science. *376*, eabi8175. https://doi.org/10.1126/science.abi8175.
3. Iqbal, N., and Iqbal, N. (2014). Imatinib: a breakthrough of targeted therapy in cancer. Chemother. Res. Pract. *2014*, 357027.
4. O'Brien, S.G., Guilhot, F., Larson, R.A., Gathmann, I., Baccarani, M., Cervantes, F., Cornelissen, J.J., Fischer, T., Hochhaus, A., Hughes, T., et al. (2003). Imatinib compared with interferon and low-dose cytarabine for newly diagnosed chronic-phase chronic myeloid leukemia. N. Engl. J. Med. *348*, 994–1004.
5. Druker, B.J., Sawyers, C.L., Kantarjian, H., Resta, D.J., Reese, S.F., Ford, J.M., Capdeville, R., and Talpaz, M. (2001). Activity of a specific inhibitor of the BCR-ABL tyrosine kinase in the blast crisis of chronic myeloid leukemia and acute lymphoblastic leukemia with the Philadelphia chromosome. N. Engl. J. Med. *344*, 1038–1042.
6. Rosenberg, S.A. (2021). A Journey in Science: Immersion in the search for effective cancer immunotherapies. Mol. Med. *27*, 63.
7. Leidner, R., Sanjuan Silva, N., Huang, H., Sprott, D., Zheng, C., Shih, Y.-P., Leung, A., Payne, R., Sutcliffe, K., Cramer, J., et al. (2022). Neoantigen T-Cell Receptor Gene Therapy in Pancreatic Cancer. N. Engl. J. Med. *386*, 2112–2119.
8. Ott, P.A., Hu, Z., Keskin, D.B., Shukla, S.A., Sun, J., Bozym, D.J., Zhang, W., Luoma, A., Giobbie-Hurder, A., Peter, L., et al. (2017). An immunogenic personal neoantigen vaccine for patients with melanoma. Nature *547*, 217–221.
9. Hu, Z., Leet, D.E., Allesøe, R.L., Oliveira, G., Li, S., Luoma, A.M., Liu, J., Forman, J., Huang, T., Iorgulescu, J.B., et al. (2021). Personal neoantigen vaccines induce persistent memory T cell responses and epitope spreading in patients with melanoma. Nat. Med. *27*, 515–525.
10. Bauman, J., Burris, H., Clarke, J., Patel, M., Cho, D., Gutierrez, M., Julian, R., Scott, A., Cohen, P., Frederick, J., et al. (2020). 798 Safety, tolerability, and immunogenicity of mRNA-4157 in combination with pembrolizumab in subjects with unresectable solid tumors (KEYNOTE-603): an update. J. Immunother. Cancer *8*. https://doi.org/10.1136/jitc-2020-SITC2020.0798.
11. Goff, S.L., Dudley, M.E., Citrin, D.E., Somerville, R.P., Wunderlich, J.R., Danforth, D.N., Zlott, D.A., Yang, J.C., Sherry, R.M., Kammula, U.S., et al. (2016). Randomized, Prospective Evaluation Comparing Intensity of Lymphodepletion Before Adoptive Transfer of Tumor-Infiltrating Lymphocytes for Patients With Metastatic Melanoma. J. Clin. Oncol. *34*, 2389–2397.
12. Lu, S.X., De Neef, E., Thomas, J.D., Sabio, E., Rousseau, B., Gigoux, M., Knorr, D.A., Greenbaum, B., Elhanati, Y., Hogg, S.J., et al. (2021). Pharmacologic modulation of RNA splicing enhances anti-tumor immunity. Cell *184*, 4032–4047.e31.
13. MacKay, M., Afshinnekoo, E., Rub, J., Hassan, C., Khunte, M., Baskaran, N., Owens, B., Liu, L., Roboz, G.J., Guzman, M.L., et al. (2020). The therapeutic landscape for cells engineered with chimeric antigen receptors. Nat. Biotechnol. *38*, 233–244.
14. Leko, V., and Rosenberg, S.A. (2020). Identifying and Targeting Human Tumor Antigens for T Cell-Based Immunotherapy of Solid Tumors. Cancer Cell *38*, 454–472.
15. Parker, K.R., Migliorini, D., Perkey, E., Yost, K.E., Bhaduri, A., Bagga, P., Haris, M., Wilson, N.E., Liu, F., Gabunia, K., et al. (2020). Single-Cell Analyses Identify Brain Mural Cells Expressing CD19 as Potential Off-Tumor Targets for CAR-T Immunotherapies. Cell *183*, 126–142.e17.
16. Shraim, R., Mooney, B., Conkrite, K.L., Weiner, A.K., Morin, G.B., Sorensen, P.H., Maris, J.M., Diskin, S.J., and Sacan, A. (2024). IMMUNOTAR - Integrative prioritization of cell surface targets for cancer immunotherapy. Preprint at bioRxiv. https://doi.org/10.1101/2024.06.04.597422.
17. Zhao, S., Zhang, Y., Gordon, W., Quan, J., Xi, H., Du, S., von Schack, D., and Zhang, B. (2015). Comparison of stranded and non-stranded

CellPress
OPEN ACCESS

RNA-seq transcriptome profiling and investigation of gene overlap. BMC Genom. *16*, 675.

18. Gry, M., Rimini, R., Strömberg, S., Asplund, A., Pontén, F., Uhlén, M., and Nilsson, P. (2009). Correlations between RNA and protein expression profiles in 23 human cell lines. BMC Genom. *10*, 365. https://doi.org/10.1186/1471-2164-10-365.
19. Jing, Y., Liu, Y., Li, Q., Ye, Y., Diao, L., Huang, Y., Zhou, Y., Green, M.R., Mills, G.B., and Han, L. (2021). Expression of chimeric antigen receptor therapy targets detected by single-cell sequencing of normal cells may contribute to off-tumor toxicity. Cancer Cell *39*, 1558–1559.
20. Love, M.I., Huber, W., and Anders, S. (2014). Moderated estimation of fold change and dispersion for RNA-seq data with DESeq2. Genome Biol. *15*, 550.
21. Robinson, M.D., McCarthy, D.J., and Smyth, G.K. (2010). edgeR: a Bioconductor package for differential expression analysis of digital gene expression data. Bioinformatics *26*, 139–140.
22. Ritchie, M.E., Phipson, B., Wu, D., Hu, Y., Law, C.W., Shi, W., and Smyth, G.K. (2015). limma powers differential expression analyses for RNA-sequencing and microarray studies. Nucleic Acids Res. *43*, e47.
23. Hastie, T., and Tibshirani, R. (1986). Generalized additive models. Stat. Sci. *1*, 297–318.
24. Mancl, L.A., and DeRouen, T.A. (2001). A covariance estimator for GEE with improved small-sample properties. Biometrics *57*, 126–134.
25. Zeger, S.L., and Diggle, P.J. (1994). Semiparametric models for longitudinal data with application to CD4 cell numbers in HIV seroconverters. Biometrics *50*, 689–699.
26. Blei, D.M., Kucukelbir, A., and McAuliffe, J.D. (2017). Variational Inference: A Review for Statisticians. J. Am. Stat. Assoc. *112*, 859–877. https://doi.org/10.1080/01621459.2017.1285773.
27. Lopez, R., Regier, J., Cole, M.B., Jordan, M.I., and Yosef, N. (2018). Deep generative modeling for single-cell transcriptomics. Nat. Methods *15*, 1053–1058.
28. Gayoso, A., Steier, Z., Lopez, R., Regier, J., Nazor, K.L., Streets, A., and Yosef, N. (2021). Joint probabilistic modeling of single-cell multi-omic data with totalVI. Nat. Methods *18*, 272–282.
29. Büttner, M., Ostner, J., Müller, C.L., Theis, F.J., and Schubert, B. (2021). scCODA is a Bayesian model for compositional single-cell data analysis. Nat. Commun. *12*, 1–10.
30. Kleshchevnikov, V., Shmatko, A., Dann, E., Aivazidis, A., King, H.W., Li, T., Elmentaite, R., Lomakin, A., Kedlian, V., Gayoso, A., et al. (2022). Cell2location maps fine-grained cell types in spatial transcriptomics. Nat. Biotechnol. *40*, 661–671.
31. Bingham, E., Chen, J.P., Jankowiak, M., Obermeyer, F., Pradhan, N., Karaletsos, T., Singh, R., Szerlip, P., Horsfall, P., and Goodman, N.D. (2018). Pyro: Deep Universal Probabilistic Programming. https://doi.org/10.48550/arXiv.1810.09538.
32. Paszke, A., Gross, S., Massa, F., Lerer, A., Bradbury, J., Chanan, G., Killeen, T., Lin, Z., Gimelshein, N., Antiga, L., et al. (2019). PyTorch: An Imperative Style, High-Performance Deep Learning Library. Adv. Neural Inf. Process. Syst. *32*.
33. Lüleci, H.B., and Yılmaz, A. (2022). Robust and rigorous identification of tissue-specific genes by statistically extending tau score. BioData Min. *15*, 31. https://doi.org/10.1186/s13040-022-00315-9.
34. Suri, A., Jagadish, N., Saini, S., and Gupta, N. (2015). Targeting cancer testis antigens for biomarkers and immunotherapy in colorectal cancer: Current status and challenges. World J. Gastrointest. Oncol. *7*, 492–502.
35. Jakobsen, M.K., and Gjerstorff, M.F. (2020). CAR T-Cell Cancer Therapy Targeting Surface Cancer/Testis Antigens. Front. Immunol. *11*, 1568.
36. Su, Y., Zhang, X., Bidlingmaier, S., Behrens, C.R., Lee, N.-K., and Liu, B. (2020). ALPPL2 is a highly specific and targetable tumor cell surface antigen. Cancer Res. *80*, 4552–4564.
37. Singh, P., Toom, S., and Huang, Y. (2017). Anti-claudin 18.2 antibody as new targeted therapy for advanced gastric cancer. J. Hematol. Oncol. *10*, 105.
38. Boitreaud, J., Mallet, V., Oliver, C., and Waldispühl, J. (2020). OptiMol: Optimization of Binding Affinities in Chemical Space for Drug Discovery. J. Chem. Inf. Model. *60*, 5658–5666.
39. Press, B. (2011). Optimization of the Caco-2 permeability assay to screen drug compounds for intestinal absorption and efflux. Methods Mol. Biol. *763*, 139–154. https://doi.org/10.1007/978-1-61779-191-8_9.
40. Yarmarkovich, M., Marshall, Q.F., Warrington, J.M., Premaratne, R., Farrel, A., Groff, D., Li, W., di Marco, M., Runbeck, E., Truong, H., et al. (2023). Targeting of intracellular oncoproteins with peptide-centric CARs. Nature *623*, 820–827.
41. Morgan, R.A., Yang, J.C., Kitano, M., Dudley, M.E., Laurencot, C.M., and Rosenberg, S.A. (2010). Case report of a serious adverse event following the administration of T cells transduced with a chimeric antigen receptor recognizing ERBB2. Mol. Ther. *18*, 843–851.
42. Conn, P., Johnson, D., Williams, P., Melin, S., and Hooten, M. (2017). A guide to Bayesian model checking for ecologists. Preprint at PeerJ. https://doi.org/10.7287/peerj.preprints.3390v1.
43. Mao, Y., Fan, W., Hu, H., Zhang, L., Michel, J., Wu, Y., Wang, J., Jia, L., Tang, X., Xu, L., et al. (2019). MAGE-A1 in lung adenocarcinoma as a promising target of chimeric antigen receptor T cells. J. Hematol. Oncol. *12*, 106–118.
44. Tang, Z., Li, C., Kang, B., Gao, G., Li, C., and Zhang, Z. (2017). GEPIA: a web server for cancer and normal gene expression profiling and interactive analyses. Nucleic Acids Res. *45*, W98–W102.
45. de Lagausie, P., Berrebi, D., Michon, J., Philippe-Chomette, P., El Ghoneimi, A., Garel, C., Brisse, H., Peuchmaur, M., and Aigrain, Y. (2003). Laparoscopic adrenal surgery for neuroblastomas in children. J. Urol. *170*, 932–935. https://doi.org/10.1097/01.ju.0000081415.49550.01.
46. Feng, Z., He, X., Zhang, X., Wu, Y., Xing, B., Knowles, A., Shan, Q., Miller, S., Hojnacki, T., Ma, J., et al. (2022). Potent suppression of neuroendocrine tumors and gastrointestinal cancers by CDH17CAR T cells without toxicity to normal tissues. Nat. Can. (Ott.) *3*, 581–594. https://doi.org/10.1038/s43018-022-00344-7.
47. Taylor, A.W. (2009). Ocular immune privilege. Eye *23*, 1885–1889.
48. Liu, R., Cheng, Q., Kang, L., Wang, E., Li, Y., Zhang, J., Xiao, H., Zhang, Y., Chu, L., Chen, X., et al. (2022). CD19 or CD20 CAR T Cell Therapy Demonstrates Durable Antitumor Efficacy in Patients with Central Nervous System Lymphoma. Hum. Gene Ther. *33*, 318–329. https://doi.org/10.1089/hum.2021.249.
49. Shadman, M., Gopal, A.K., Smith, S.D., Lynch, R.C., Ujjani, C.S., Turtle, C.J., Greenbaum, A., Redman, M., Yeung, C., Sersch, M., et al. (2019). CD20 Targeted CAR-T for High-Risk B-Cell Non-Hodgkin Lymphomas. Blood *134*. https://doi.org/10.1182/blood-2019-125102.
50. Song, X., Guo, C., Zheng, Y., Wang, Y., Jin, Z., and Yin, Y. (2018). Post-transcriptional regulation of cancer/testis antigen MAGEC2 expression by TRIM28 in tumor cells. BMC Cancer *18*, 971.
51. Conteduca, G., Fenoglio, D., Parodi, A., Battaglia, F., Kalli, F., Negrini, S., Tardito, S., Ferrera, F., Salis, A., Millo, E., et al. (2016). AIRE polymorphism, melanoma antigen-specific T cell immunity, and susceptibility to melanoma. Oncotarget *7*, 60872–60884.
52. Lucas, S., De Plaen, E., and Boon, T. (2000). MAGE-B5, MAGE-B6, MAGE-C2, and MAGE-C3: four new members of the MAGE family with tumor-specific expression. Int. J. Cancer *87*, 55–60.
53. Costanzo, V., Bardelli, A., Siena, S., and Abrignani, S. (2018). Exploring the links between cancer and placenta development. Open Biol. *8*, 180081. https://doi.org/10.1098/rsob.180081.
54. Blazer, L.L., Lima-Fernandes, E., Gibson, E., Eram, M.S., Loppnau, P., Arrowsmith, C.H., Schapira, M., and Vedadi, M. (2016). PR Domain-containing Protein 7 (PRDM7) Is a Histone 3 Lysine 4 Trimethyltransferase. J. Biol. Chem. *291*, 13509–13519.

55. Escobar-Hoyos, L., Knorr, K., and Abdel-Wahab, O. (2019). Aberrant RNA Splicing in Cancer. Annu. Rev. Cell Biol. *3*, 167–185.

56. Xiong, Y., Deng, Y., Wang, K., Zhou, H., Zheng, X., Si, L., and Fu, Z. (2018). Profiles of alternative splicing in colorectal cancer and their clinical significance: A study based on large-scale sequencing data. EBioMedicine *36*, 183–195.

57. Chai, S., Smith, C.C., Kochar, T.K., Hunsucker, S.A., Beck, W., Olsen, K.S., Vensko, S., Glish, G.L., Armistead, P.M., Prins, J.F., and Vincent, B.G. (2022). NeoSplice: a bioinformatics method for prediction of splice variant neoantigens. Bioinform. Adv. *2*, vbac032.

58. Sheffer, M., Lowry, E., Beelen, N., Borah, M., Amara, S.N.-A., Mader, C.C., Roth, J.A., Tsherniak, A., Freeman, S.S., Dashevsky, O., et al. (2021). Genome-scale screens identify factors regulating tumor cell responses to natural killer cells. Nat. Genet. *53*, 1196–1206.

59. Madan, S., Sinha, S., Gutkind, S.J., Cohen, E.E.W., Schäffer, A.A., and Ruppin, E. (2023). Pan-cancer analysis of patient tumor single-cell transcriptomes identifies promising selective and safe CAR targets in head and neck cancer. Preprint at bioRxiv. https://doi.org/10.1101/2021.09.29.462485.

60. Joshi, S., and Durden, D.L. (2019). Combinatorial Approach to Improve Cancer Immunotherapy: Rational Drug Design Strategy to Simultaneously Hit Multiple Targets to Kill Tumor Cells and to Activate the Immune System. JAMA Oncol. *2019*, 5245034. https://doi.org/10.1155/2019/5245034.

61. Savanur, M.A., Weinstein-Marom, H., and Gross, G. (2021). Implementing Logic Gates for Safer Immunotherapy of Cancer. Front. Immunol. *12*, 780399. https://doi.org/10.3389/fimmu.2021.780399.

62. Roybal, K.T., Williams, J.Z., Morsut, L., Rupp, L.J., Kolinko, I., Choe, J.H., Walker, W.J., McNally, K.A., and Lim, W.A. (2016). Engineering T Cells with Customized Therapeutic Response Programs Using Synthetic Notch Receptors. Cell *167*, 419–432.e16.

63. Kloss, C.C., Condomines, M., Cartellieri, M., Bachmann, M., and Sadelain, M. (2013). Combinatorial antigen recognition with balanced signaling promotes selective tumor eradication by engineered T cells. Nat. Biotechnol. *31*, 71–75.

64. Tousley, A.M., Rotiroti, M.C., Labanieh, L., Rysavy, L.W., Kim, W.-J., Lareau, C., Sotillo, E., Weber, E.W., Rietberg, S.P., Dalton, G.N., et al. (2023). Co-opting signalling molecules enables logic-gated control of CAR T cells. Nature *615*, 507–516.

65. Zhou, S., Liu, M., Ren, F., Meng, X., and Yu, J. (2021). The landscape of bispecific T cell engager in cancer treatment. Biomark. Res. *9*, 38.

66. Ghasemi, S. (2020). Cancer's epigenetic drugs: where are they in the cancer medicines? Nature *20*, 367–379. https://doi.org/10.1038/s41397-019-0138-5.

67. Malaker, S.A., Penny, S.A., Steadman, L.G., Myers, P.T., Loke, J.C., Raghavan, M., Bai, D.L., Shabanowitz, J., Hunt, D.F., and Cobbold, M. (2017). Identification of glycopeptides as post-translationally modified neoantigens in leukemia. Cancer Immunol. Res. *5*, 376–384.

68. Thul, P.J., and Lindskog, C. (2018). The human protein atlas: A spatial map of the human proteome. Protein Sci. *27*, 233–244.

69. Li, G., Mahajan, S., Ma, S., Jeffery, E.D., Zhang, X., Bhattacharjee, A., Venkatasubramanian, M., Weirauch, M.T., Miraldi, E.R., Grimes, H.L., et al. (2024). Splicing neoantigen discovery with SNAF reveals shared targets for cancer immunotherapy. Sci. Transl. Med. *16*, eade2886. https://doi.org/10.1126/scitranslmed.ade2886.

70. Yan, X., Hu, S., Mao, Y., Ye, Y., and Yu, H. (2021). Deep multi-view learning methods: A review. Neurocomputing *448*, 106–129. https://doi.org/10.1016/j.neucom.2021.03.090.

71. GTEx Consortium (2020). The GTEx Consortium atlas of genetic regulatory effects across human tissues. Science *369*, 1318–1330.

72. Cancer Genome Atlas Research Network; Weinstein, J.N., Collisson, E.A., Mills, G.B., Shaw, K.R.M., Ozenberger, B.A., Ellrott, K., Shmulevich, I., Sander, C., and Stuart, J.M. (2013). The Cancer Genome Atlas Pan-Cancer analysis project. Nat. Genet. *45*, 1113–1120.

73. Tate, J.G., Bamford, S., Jubb, H.C., Sondka, Z., Beare, D.M., Bindal, N., Boutselakis, H., Cole, C.G., Creatore, C., Dawson, E., et al. (2019). COSMIC: the Catalogue Of Somatic Mutations In Cancer. Nucleic Acids Res. *47*, D941–D947. https://doi.org/10.1093/nar/gky1015.

74. Emig, D., Salomonis, N., Baumbach, J., Lengauer, T., Conklin, B.R., and Albrecht, M. (2010). AltAnalyze and DomainGraph: analyzing and visualizing exon expression data. Nucleic Acids Res. *38*, W755–W762.

75. Garrido-Martín, D., Palumbo, E., Guigó, R., and Breschi, A. (2018). ggsashimi: Sashimi plot revised for browser- and annotation-independent splicing visualization. PLoS Comput. Biol. *14*, e1006360.

76. Dobin, A., Davis, C.A., Schlesinger, F., Drenkow, J., Zaleski, C., Jha, S., Batut, P., Chaisson, M., and Gingeras, T.R. (2013). STAR: ultrafast universal RNA-seq aligner. Bioinformatics *29*, 15–21.

77. Uhlén, M., Fagerberg, L., Hallström, B.M., Lindskog, C., Oksvold, P., Mardinoglu, A., Sivertsson, Å., Kampf, C., Sjöstedt, E., Asplund, A., et al. (2015). Tissue-based map of the human proteome. Science *347*, 1260419.

78. Kingma, D.P., and Ba, J. (2014). Adam: A Method for Stochastic Optimization. Preprint at arXiv. https://doi.org/10.48550/arXiv.1412.6980.

79. Almeida, L.G., Sakabe, N.J., deOliveira, A.R., Silva, M.C.C., Mundstein, A.S., Cohen, T., Chen, Y.-T., Chua, R., Gurung, S., Gnjatic, S., et al. (2009). CTdatabase: a knowledge-base of high-throughput and curated data on cancer-testis antigens. Nucleic Acids Res. *37*, D816–D819.

80. Saito, T., and Rehmsmeier, M. (2015). The Precision-Recall Plot Is More Informative than the ROC Plot When Evaluating Binary Classifiers on Imbalanced Datasets. PLoS One *10*, e0118432.

CellPress
OPEN ACCESS

STAR★METHODS

KEY RESOURCES TABLE

REAGENT or RESOURCE	SOURCE	IDENTIFIER
Deposited data		
GTEx Gene TPM data	The GTEx Consortium[71]	https://gtexportal.org/home/downloads/adult-gtex/bulk_tissue_expression
GTEx Raw Fastq data	The GTEx Consortium[71]	phs000424.v8.p2
TCGA Raw Fastq data	The Cancer Genome Atlas Research Network[72]	phs000178.v11.p8
TCGA SKCM TPM data	The Cancer Genome Atlas Research Network[72]	https://xenabrowser.net/datapages/
Human Protein Atlas protein staining data	Thul et al.[68]	https://www.proteinatlas.org/about/download
COSMIC somatic mutation	Tate et al.[73]	https://cancer.sanger.ac.uk/cosmic/download
Tumor-specific antigen benchmark datasets	This paper	https://www.synapse.org/Synapse:syn61670083
Software and algorithms		
BayesTS	This paper	https://github.com/frankligy/BayesTS
Source code	This paper	https://doi.org/10.5281/zenodo.13922316
AltAnalyze v2.1.4	Emig et al.[74]	https://www.altanalyze.org/
ggsashimi v1.1.5	Garrido-Martín et al.[75]	https://github.com/guigolab/ggsashimi
STAR v2.4.0h	Dobin et al.[76]	https://github.com/alexdobin/STAR
Pyro v1.8.4	Bingham et al.[31]	https://pyro.ai/

METHOD DETAILS

Datasets used in the work

De-identified human sample gene expression, protein and splicing profiles were derived from previously published cohorts and studies. The development and validation of the described algorithm was conducted entirely using publicly available datasets and in silico methods.

BayesTS model

BayesTS was developed as a hierarchical Bayesian model that jointly considers multiple types of molecular measurements that independently quantifies tumor specificity for a target. In our model, we consider three separate observations, namely, tissue distribution of the bulk RNA-Seq counts (**X**), normalized bulk RNA-Seq counts (**Y**) and protein-level expression derived from immunohistochemistry slides (**Z**). The provided base model can potentially be extended to incorporate other modalities (e.g., single-cell cell-type distributions). We define the tumor specificity as the expression of a target in normal tissues bounded by 0 and 1, where a smaller value should represent a more confident potential target (e.g., tumor specific) where a higher value represents greater risk of being non-specific (Figure 1A). Non-diseased tissue bulk RNA-Seq data was obtained from GTEx and TCGA matched controls (see bulk RNA-seq preprocessing), and the normal tissue protein immunohistochemistry annotations were downloaded from Human Protein Atlas (HPA)[77] (see immunohistochemistry preprocessing).

Here, we denote the expression level in normal tissues (tumor specificity) as σ and assume all observations are generated by this underlying latent variable through a hierarchical sampling process. Specifically, the shape and values of the three observations are explained below. A tissue distribution observation $\mathbf{X_{it}}$, where **i** denotes a given target and **t** denotes a tissue type, **i** can be any druggable target (e.g., gene, protein, splice junction, MHC-presented neoantigen, mutation, etc). In our default model, the total number of tissues considered is **T**, where each value in the matrix **X** represents the percentage of samples in each tissue type in which this target can be detected. A Normalized bulk RNA-Seq observation $\mathbf{Y_{is}}$, where **s** denotes a normal sample in our model and the total number of samples considered is **S** and each value in matrix **Y** represents the normalized count value (Transcript Per Million (TPM)) for each target in the sample. Last but not least, the protein annotation observation is defined as $\mathbf{Z_{ip}}$, where **p** denotes the number of immunohistochemistry slides for a target. In our model, the total number of slides considered is **P**. Each value in matrix **Z** represents a

categorical value {0, 1, 2, 3} which corresponds to the HPA consortium annotations {high, medium, low, not detected}. The plate notation for this hierarchical model is shown in Figure S4.

Mathematically, the model can be described as the following. First, since the inferred tumor specificity (σ) is strictly bounded by 0 and 1, we use a Beta distribution to represent this latent variable with a weakly informative prior Beta(2,2), which is symmetric and centers the distribution at 0.5. This prior places approximately 80% of the probability mass within the interval (0.2, 0.8), while limiting the mass below 0.1 (indicating tumor-specific genes) to only 3%. Such a choice ensures the prior is weakly informative, allowing the model to primarily learn tumor specificity from the data, rather than being overly influenced by the prior assumptions. We conducted sensitivity tests using different Beta specifications (Figures S5A–S5C) and have shown the results are robust to the alpha and beta parameters and both are tunable parameters in the model.

$$\sigma_i \sim Beta(2,2) \quad \text{(Equation 1)}$$

The normalized count value (**Y**) is non-negative and is modeled by a LogNormal distribution with a fixed scale parameter 0.5, whereas the loc parameter is defined by the underlying tumor specificity σ. The intuition is if a target is highly expressed (tumor specificity σ skewed toward 1) then it is highly likely to have a higher mean value for the normalized count value. We use a fixed variance because we do not observe overdispersion and it is also advisable to begin with a low variance model for better training convergence. Another property of LogNormal distribution is the magnitude of values increasing exponentially, which coincide with the normalized RNA count data. We introduce a scale factor β_{y_i} to compensate the different magnitudes between σ and the observed value in **Y.** We first adopted an empirical Bayesian approach to estimate the optimal coefficient between BayesTS sigma and observed normalized count under the assumption of the lognormal distribution. Particularly, we first calculated the empirical mean of each gene normalized count (denote as mean). The presumed lognormal distribution allows us to derive the mean parameter of the lognormal distribution for each gene from the actual mean in the observed gene count data as $\mu_g = e^{mean_g} - \frac{0.5^2}{2}$. We then took 100 genes as anchor points along the percentile (1th percent, 2th percent, … 100th percent) and fit a linear regression model to estimate the magnitude between the sigma (bound by 0 and 1) and μ_g (Figures S5D–S5F) and we denote the inferred coefficient as y_{ebayes}.

$$\beta_{y_i} \sim Gamma(y\_ebayes, 1) \quad \text{(Equation 2)}$$

$$Y_{is} \sim LogNormal\left(\beta_{y_i} \times \sigma, 0.5\right) \quad \text{(Equation 3)}$$

The tissue distribution is to describe RNA expression across different tissues. The input **X** to the model represents the percentage of samples expressing this target with each tissue. Most of the curated tissue types in our reference set have a minimum number of 25 samples (sampled GTEx dataset of matching donors) but increase to hundreds of samples for some tissues (associated with common cancers), we chose 25 as the total count, to transform the ratio data to count data, so that we can model it using Poisson distribution. Similar to the normalized count value, we use β_{x_i} to account for the difference in magnitude between σ and the observed value in **X**. Here we use 25 as the mean of the scaling factor, because it assumes a target expressed at a medium level should have ~12.5 samples (half), expressed in each tissue.

$$\beta_{x_i} \sim Gamma(25, 1) \quad \text{(Equation 4)}$$

$$scaled\ X_{it} = 25 \times X_{it} \quad \text{(Equation 5)}$$

$$scaled\ X_{it} \sim Poisson\left(\beta_{x_i} \times \sigma\right) \quad \text{(Equation 6)}$$

Here we allow the user to tune the tissue importance by introducing a weight vector $w_t \in [0, 1]$, by default, all the values in the tissue weight vector are 0.5, representing equal importance for each tissue. This weight vector is incorporated as the probability parameter in a binomial distribution. We introduce the random variable **Total** that denotes the total number of samples for each tissue:

$$Total_t \sim Binomial(50, w_t) \quad \text{(Equation 7)}$$

By default, all weights are 0.5, so that the total count for each tissue should be ~25, which is the same as the vanilla model defined above. However, when users have a strong reason to downweight or upweight certain tissues, w_t becomes important. For example, if we adjust the importance of tonsil to 0.1, then the total count that can be distributed is only around 5 instead of 25 for the tonsil samples. In an extreme case, even if the target is expressed in 90% of tonsil samples, the actual scaled count value the model considers is only 4.5, which won't affect the model inference as much as it could in default setting.

For the protein data, each entry in the matrix **Z** is assigned a categorical value of 0, 1, 2 and 3 (highly, medium, low and no expression, respectively - see immunohistochemistry preprocessing). We model the observation **Z** as a categorical distribution where the probability for observing a label as high, medium, low and not detected is derived from the underlying parameter σ. Particularly, when

the value of σ is high, the chance of observing the label as high is also increased, and vice versa. Hence, we parameterize the probability vector in the categorical distribution as following:

$$P(high) = \frac{2}{3}\sigma \qquad \text{(Equation 8)}$$

$$P(medium) = \frac{1}{3}\sigma \qquad \text{(Equation 9)}$$

$$P(low) = \frac{1}{3}(1-\sigma) \qquad \text{(Equation 10)}$$

$$P(Not\ detected) = \frac{2}{3}(1-\sigma) \qquad \text{(Equation 11)}$$

$$Z_{ip} \sim Categorical(\ p(high), p(medium), p(low), p(Not\ detected)\ ) \qquad \text{(Equation 12)}$$

Although the tissue importance is mainly used for tuning the tissue distribution (**X**), we also populate the weight vector w_t to the protein level to augment the tuning effect (when protein data is available for inference). Here, the weights impact the number of "high" and "not detected" labels in the input **Z** matrix. Specifically, if we denote the original protein expression label distribution of gene i in tissue t as $Z_{it} \in \{0,1,2,3\}$ and the number of high, medium, low and not detected are n_0, n_1, n_2, n_3. Given the weight, the updated numbers can be derived as following:

We first derive a scaling factor for each tissue sf_t based on its ratio against the baseline:

$$sf_t = \begin{cases} \frac{w_t}{0.5} & wt < 0.5 \\ \frac{w_t}{0.5} - 1 & wt > 0.5 \end{cases}$$

If $w_t < 0.5$:

$$n_l(updated) \sim Binomial(|n_l|, sf_t) \quad where\ l = 0,1,2 \qquad \text{(Equation 13)}$$

$$n_{\{3\}}(updated) = \sum_{l=0}^{2} Binomial(|n_l|, 1 - sf_t) + n_3 \qquad \text{(Equation 14)}$$

Elif $w_t > 0.5$:

$$n_l(updated) \sim Binomial(|n_l|, 1 - sf_t) \quad where\ l = 1,2,3 \qquad \text{(Equation 15)}$$

$$n_{\{0\}}(updated) = \sum_{l=1}^{3} Binomial(|n_l|, sf_t) + n_0 \qquad \text{(Equation 16)}$$

The intuition is, for instance, when the weight of tonsil becomes 0.1, the number of "high", "medium" and "low" will be reduced by replacing these assignments as "not detected" following in Bernoulli probability equal to the scaling factor 0.2 (0.1/0.5). On the other hand, if the weight is as high as 0.9, then the number of "medium", "low" and "not detected" will instead be reduced by replacing them as "high" following Bernoulli probability equal to the complement of scaling factor 0.8 (0.9/0.5–1), which arrives at 0.2. When the weight equals the default value of 0.5, no update will be performed.

Extending BayesTS to custom modalities

BayesTS can be extended to incorporate additional modalities. To demonstrate this, we applied BayesTS to identify melanoma-specific targets. The base BayesTS model primarily considers the safety profile. By incorporating the log fold change (LFC) between melanoma gene expression and normal skin, we can further account for therapeutic windows and identify genes possessing both properties. We obtained the LFC values through the GEPIA API (http://gepia.cancer-pku.cn/assets/PHP2/differential_genes.php?methodoption = limma&dataset = {cancer_shortname}). For melanoma, the cancer abbreviation is SKCM. We assume the LFC follows a normal distribution with the mean equal to the GEPIA-reported LFC value,[44] as follows:

$$LFC \sim Normal\ (lfc, 0.5) \qquad \text{(Equation 17)}$$

We sampled 100 data points from this normal distribution to serve as the data observations for this additional modality. The relationship between the underlying BayesTS σ and the mean lfc is modeled as:

$$\beta_{lfc} \sim Beta\,(2,1) \quad \text{(Equation 18)}$$

$$lfc = -log_{10}(\sigma) \times \beta_{lfc} \quad \text{(Equation 19)}$$

Since BayesTS values are bounded between 0 and 1, we adopted a log10 transformation to extend the range. Similar to the approach used for the base modalities, we applied a coefficient to account for the magnitude differences in LFC values. Typically, LFC cutoffs are 0.5, 1, and 2, which correspond to 1.5, 2, and 4-fold changes. This configuration ensures these cutoffs correspond to approximately 0.5, 0.3, and 0.1 in the resultant BayesTS score, respectively. This makes sense because a 4-fold change, which is highly significant, should result in a low score around 0.1. A 2-fold change, also notable, corresponds to a score of 0.3. A 1.5-fold change, being the minimum threshold of significance, is mapped to a score of 0.5. We also provided a tutorial on how to set up this extension on our GitHub page (https://github.com/frankligy/BayesTS/tree/main/extension).

Deriving a posterior distribution using variational inference

We derive the posterior distribution for the tumor specificity parameter σ using Variational Inference (VI) as it is faster and more scalable than Monte Carlo Markov Chain (MCMC) (Figure S5G). Briefly speaking, variational inference aims to construct a simpler distribution (variational distribution) $q_\phi(\sigma)$ that is easy to sample from, to approximate the real posterior distribution $p_\theta(\sigma|x)$ after fitting the observed data. It transforms the approximation into an optimization problem as minimizing the differences between two distributions (measured by Kullback-Leibler divergence) is equivalent to maximize the ELBO:

$$ELBO \equiv E_{q_\phi(\sigma)}\left[log\, p_\theta(x,\sigma) - log\, q_\phi(\sigma)\right] \quad \text{(Equation 20)}$$

Instead of using a simple mean-field approximation which assumes a normal distribution for each random variable and independence, we adopted a richer set of distributions for the variational distribution to resemble the true posterior to avoid bias and divergence. Specifically, we use Beta distribution to model the sigma and same Gamma distribution to model the coefficients as the model:

$$\sigma \sim Beta(alpha, beta) \quad \text{(Equation 21)}$$

$$\beta_x \sim Gamma\,(25,1) \quad \text{(Equation 22)}$$

$$\beta_y \sim Gamma\,\left(y_{ebayes,}1\right) \quad \text{(Equation 23)}$$

$$Total_t \sim Binomial(50, w_t) \quad \text{(Equation 24)}$$

Where y_{ebayes} and w_t the same as defined in the model. We train the model and maximize the ELBO using Adam optimizer[78] with a learning rate set to 0.002 and beta parameters as (0.95, 0.999). Training epoch is set to 5,000 which has been shown to achieve convergence in our experiments (Figure S1A). The learnable parameter alpha and beta from variational distribution will constitute posterior distribution for the BayesTS. Since the multiple observations inherently lie on different scales and magnitudes, to avoid the situation where one piece of molecular evidence dominates the training process, we scale the log probability of each distribution based on the empirical evidence by first training each of them. The maximum losses for each single evidence were used to determine the actual scaling factor when combining them (Figure S1A). We also recommend performing this step when the user extends our model to an internal dataset for retraining, as this will make sure that the derived scaling factor is specific to the datasets being collected.

QUANTIFICATION AND STATISTICAL ANALYSIS

Benchmark BayesTS performance

To compare the BayesTS performance in an unbiased and quantitative way, we first collected a list of tumor-specific genes including known CAR-T tumor targets, cancer testis antigens and lowly expressed genes with very restricted expression in normal tissues, respectively from CARTSC,[19] CTdatabase[79] and HPA website (annotated as either HPA_not_detected, HPA_grouped_enriched, HPA_tissue_enriched). It includes 654 CAR-T targets including the 71 in clinical trials and other predicted by previously published independent studies,[13] along with 258 curated cancer testis antigens, whose expression is restricted to testis in adult healthy tissue and usually exploited and overexpressed in tumors and 2,933 lowly expressed genes including known tumor associated genes MPO in AML, PHOX2B in neuroblastoma, CA9 in kidney cancer, etc. We then compare how the BayesTS score can prioritize such known safe targets and calculate the precision and recall in every single cutoff and result for a single Area Under Precision Recall (AUPR) level to unbiasedly compare performance. Given the unbalanced true and false hits, AUPR is more suitable than Area Under Receiver

Operating Characteristics (AUROC) value.[80] The HPA reported tissue specificity values were downloaded from the HPA web portal and only the genes with HPA reported Tau based specificity were used for benchmarking. Melanoma specific genes were downloaded from HPA with the labels tissue enriched, group enriched, not detected or tissue enhanced indicating the absence in normal tissue, along with either melanoma specific or known tumor antigens. All required code to reproduce this analysis is at https://github.com/frankligy/BayesTS/tree/main/reproduce. limma results (healthy skin only) were run by GEPIA and retrieved using GEPIA PHP API. limma results (considering all normal tissue) were run using AltAltanalyze version 2.1.4 (docker container: frankligy123/altanalyze:0.7.0.1) which implements the same eBayes approach as limma.[74] limma rank was derived from taking the ascending rank of adjusted *p*-value and descending rank of log fold change to incorporate both effect size and statistical significance.

Bulk RNA-seq preprocessing

Raw FASTQ files were downloaded from GTEx following dbGAP authorization using the ANVIL cloud platform (phs000424.v8.p2). In total 2,942 unique tissue samples corresponding to 52 non-diseased tissues were selected, ranging from 5 to 360 samples per tissue where 72% samples have around 25 samples. Tissues with less than 10 samples were excluded for tissue distribution analysis due to lack of statistical power. Genome and transcriptome alignment were performed using STAR version 2.4.0h,[76] according to TCGA recommendations, to hg38 and Gencode v36 (https://gdc.cancer.gov/about-data/gdc-data-processing/gdc-reference-files). We strictly follow the guidance of TCGA RNA-Seq manual (https://docs.gdc.cancer.gov/Data/PDF/Data_UG.pdf) as appreciable splice junction differences have been seen in our internal analysis when different STAR versions or genome annotations were used, to jointly analyze GTEx and TCGA normal controls together, in order to minimize possible batch effects. TCGA pre-aligned BAM files were downloaded from GDC portal (https://portal.gdc.cancer.gov/) for matched paratumor controls (15 additional tissues). The Gene level counts and splice junction counts were derived from AltAnalyze version 2.4.1.2 where junction counts were primarily used for all downstream analyses (gene and splicing). To reduce the impact of sequencing depth differences, we normalized the gene level and splice junction counts by either AltAnalyze junction-level RPKM values (gene length is the sum of junction lengths) or retrieved the Transcript Per Million (TPM) from the GTEx portal. The normalized counts serve as the input matrix **Y**.

We further count the number of samples in each tissue type in which a certain target is detected as expressed (see below). Although intuitively a count of zero should be considered as the cutoff for determining whether a target is detected or not, we observe diverse scenarios where very low normalized counts (less than 1) do not correspond well with the external resources for well-known tissue-specific genes (i.e., NY-ESO-1). We hypothesize choosing a cutoff to filter out the background noise would be beneficial for the purpose of better inference. We determine the cutoff by measuring the concordance between our tissue distribution against the external reference provided by Human Protein Atlas (HPA).[77] The RNA consensus tissue distribution (RNA consensus tissue gene data) was downloaded from the HPA portal (https://www.proteinatlas.org/about/download). The optimal cutoff can maximize the correspondence (Spearman r correlation and Area Under Precision Recall [AUPR]) between these two resources (Table S5). The thresholded tissue distribution profiles serve as the input matrix **X**.

TCGA Skin Cutaneous Melanoma (SKCM) bulk RNA-Seq BAM files were downloaded from NCI Genomics Data Commons and SRA, following dbGAP authorization (phs000178.v11.p8).The resultant BAM files were processed using AltAnalyze 2.1.4.2[74] to obtain the gene count matrix and splice junction matrices. Sashimi plot visualization was generated using ggsashimi package.[75]

Immunohistochemistry preprocessing

We download non-diseased tissue protein immunohistochemistry annotations (Normal tissue data) from the HPA portal (https://www.proteinatlas.org/about/download). The HPA records denote whether a protein product is present or not in a certain tissue type. The annotation contains four levels, namely high, medium, low and not detected. We used a customized script (available at https://github.com/frankligy/BayesTS/blob/main/BayesTS.py#L236) to reshape these values into a matrix of the shape (n_protein, 4) where each column corresponds to the number of each annotation level. Since each protein (gene) has a variable number of total slides annotated, we normalized each gene to the average number of slides ($p = 89$ in our data) across all available genes. Finally, we expand the number of labels into categorical values of high, medium, low and not detected are encoded as numerical values 0, 1, 2 and 3, respectively and the total columns amount to $p = 89$. Here the weight vector w_t acts by switching the annotation labels defined by the weight probability (See BayesTS model). The resultant categorical matrix serves as the input matrix **Z**.

Prior and posterior check

To assure the Bayesian model learns from the observed data in order to adjust for the prior information, we performed prior and posterior checks by simulating samples from both prior and posterior distribution and comparing these against the actual observations. The prior of tumor specificity σ is calculated as the average of 1,000 bootstraps from the randomly initiated Beta(2,2) distribution. The posterior of tumor specificity is calculated as the average of 1,000 bootstraps from the learned Beta distribution parameterized by its alpha and beta parameters in the variational distribution (guide function). For the tissue distribution, we used Poisson distribution with fixed coefficient 25 to simulate a vector of the same length for available tissue (T). For the normalized count, the empirically determined coefficient was used, as defined by BayesTS model, to simulate out a vector of the same length of all available samples (S). For the protein annotations, we sampled P labels from the underlying categorical distribution with the probabilities determined by the prior or posterior tumor specificities same as the model (See BayesTS model).

CAR-T therapy targets evaluation

We obtained a list of 71 CAR-T targets that are currently under at least one NCI-registered clinical trials from the CARTSC database[19] (https://hanlab.tamhsc.edu/CARTSC/#!/). We intersect the 71 targets with the genes that have protein level information available to obtain 54 CAR-T targets for model evaluation.

Gene enrichment and network analysis

The GO-Elite molecular interaction database in the AltAnalyze v.2.1.4 software, was used as the basis for identifying putative protein-protein and protein-DNA interactions associated with distinct BayesTS specificity bins. For these analyses, they were performed using a hypergeometric test comparing genes present within a BayesTS ranked specificity bin. Gene set enrichments were performed in the software GO-Elite considering diverse pathways, gene set, Ontology and built-in cellular biomarker databases. Cancer Gene mutation data were downloaded from COSMIC database[73] (https://cancer.sanger.ac.uk/cosmic/download) for the correlation analysis. Specifically, we extracted 12 diverse types of variants (synonymous variants, missense variants, intron variants, stop codon lost, stop codon gained, splice 3′ UTR, splice 5′ UTR, splice region variants, splicing acceptor variants, splicing donor variants, in-frame deletion, in-frame insertion), along with the copy number variants (increase or decrease) and differential methylation states (hypermethylation and hypomethylation). Manually-annotated variants were aggregated into gene level and normalized into the range of 0–1 to circumvent the bias that certain genes were more well-studied than others. We correlated BayesTS score and the normalized variants score on common genes present in the variant list using Pearson correlation.

Cell Reports Methods, Volume *4*

Supplemental information

Quantifying tumor specificity using Bayesian probabilistic modeling for drug and immunotherapeutic target discovery

Guangyuan Li, Daniel Schnell, Anukana Bhattacharjee, Mark Yarmarkovich, and Nathan Salomonis

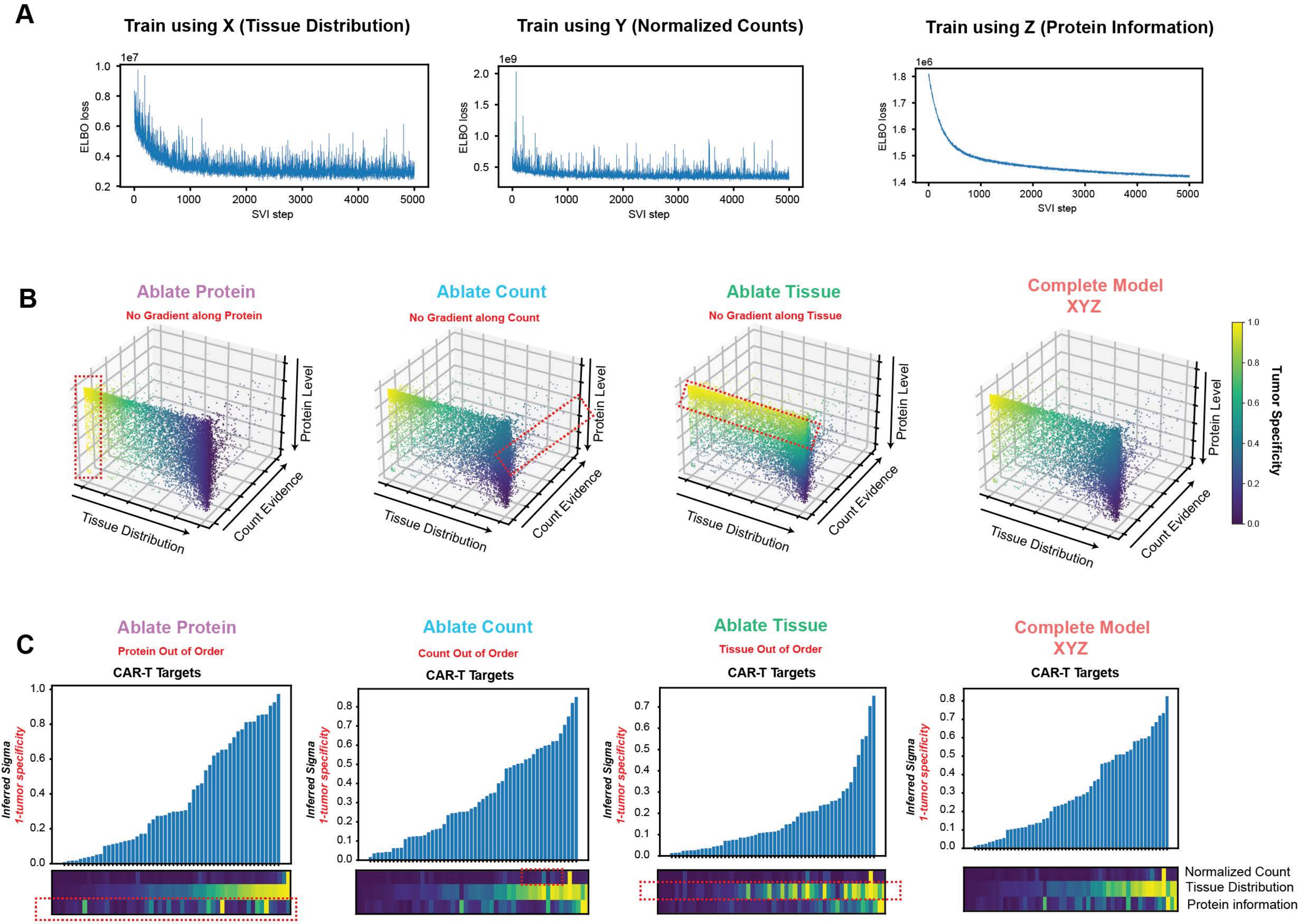

Supplementary Figure 1. BayesTS incorporates multiple data dimensions for inference. A) The Evidence Lower Bound (ELBO) loss across the training process when only one evidence is provided. B,C) Ablation test on each evidence and the exhaustive combinations of all forms of evidence for all 13,306 protein coding genes (B) and 54 CAR-T targets in clinical trials, the color corresponding to tumor specificity value, where higher values means highly tumor-specific or absent in normal tissue, and vice versa C) From left to right are X and Y, X and Z, Y and Z, X, Y and Z. Red box highlights the impact when each modality is left out. Y-axis indicates the BayesTS inferred sigma, which is the complement of tumor specificity score shown in B (1-sigma). Related to Figure 1 and 2.

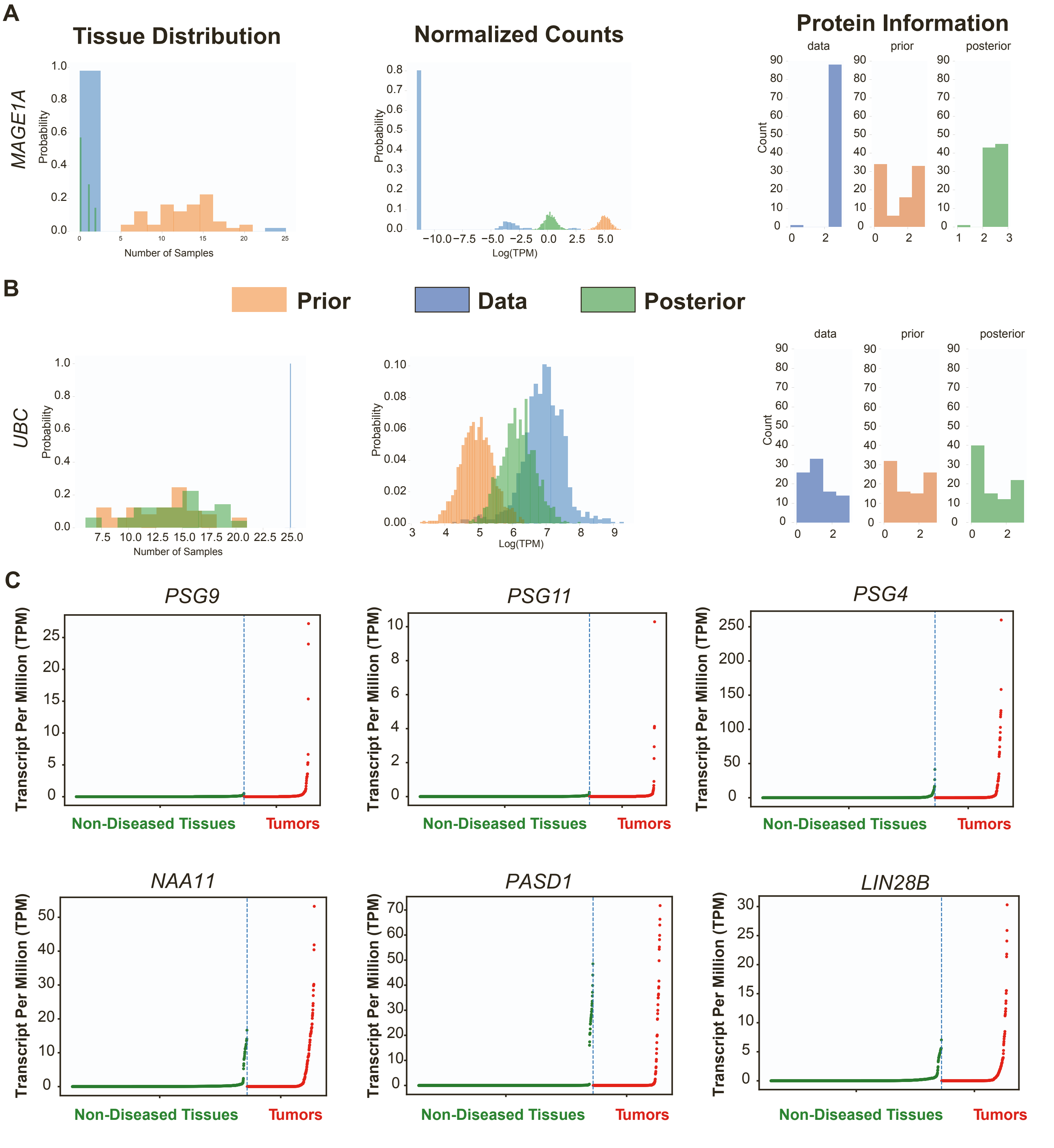

Supplementary Figure 2. Prior and Posterior check for the BayesTS inference process for two representative targets, along with novel drug targets using BayesTS A) A lowly expressed target, MAGE1A. B) A highly expressed target, UBC. From left to right, each panel represents one form of evidence, namely, tissue distribution X, normalized RNA count Y and protein-level information Z. Orange represents the prior distribution, blue the observed actual information and green the posterior distribution after the Bayesian variational inference. (C) Tissue-specificity RNA expression plots for control tissues (green) and melanoma patient samples (red), where each point is an independent sample (blue lines separating normal and tumor tissue samples, organized by tissue type). Examples are shown for Pregnancy-specific glycoproteins (PSG), namely PSG6, PSG9 and PSG4 and other potentially novel targets for therapy. Related to Figure 2 and 3.

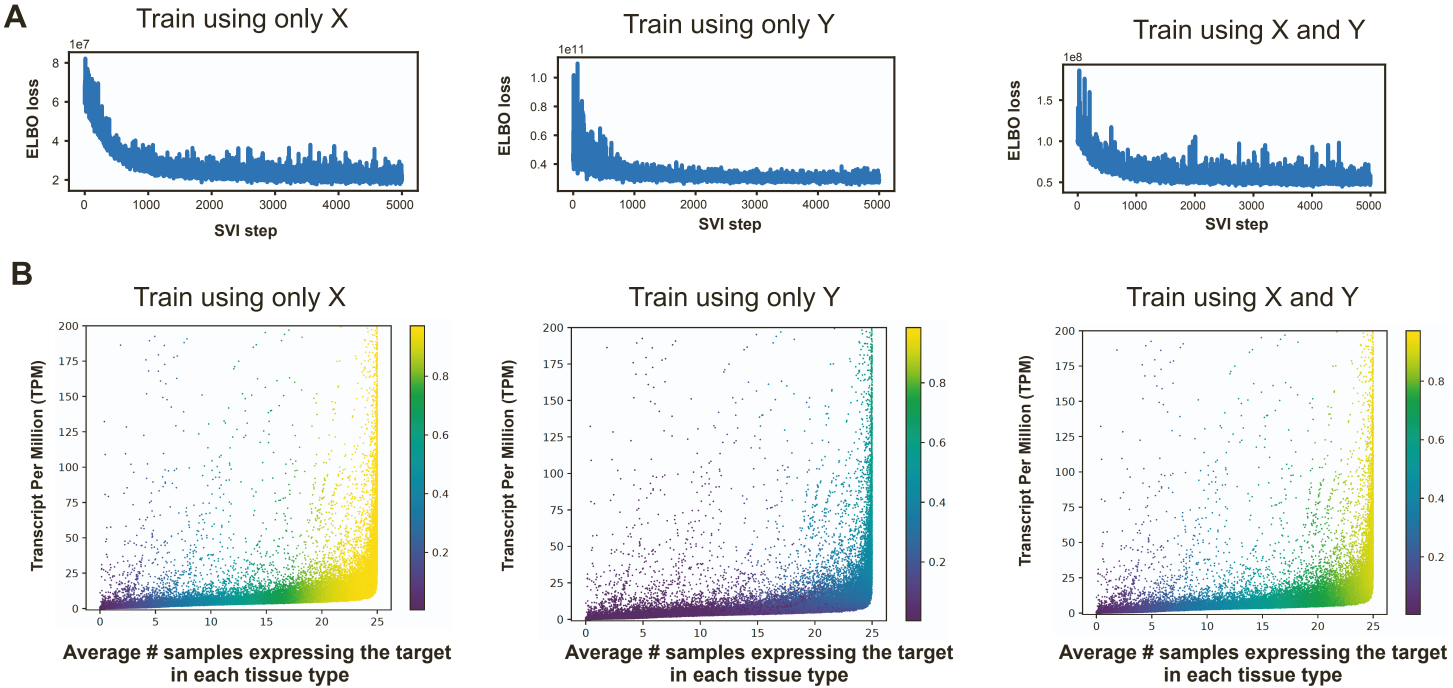

Supplementary Figure 3. Training loss and validation of BayesTS on splice junction targets. A) The ELBO loss of training tissue distribution (X) or normalized RNA count (Y) separately and together (right). B) The inferred tumor specificity scores projected on both X and Y axis in three different scenarios. Related to Figure 3.

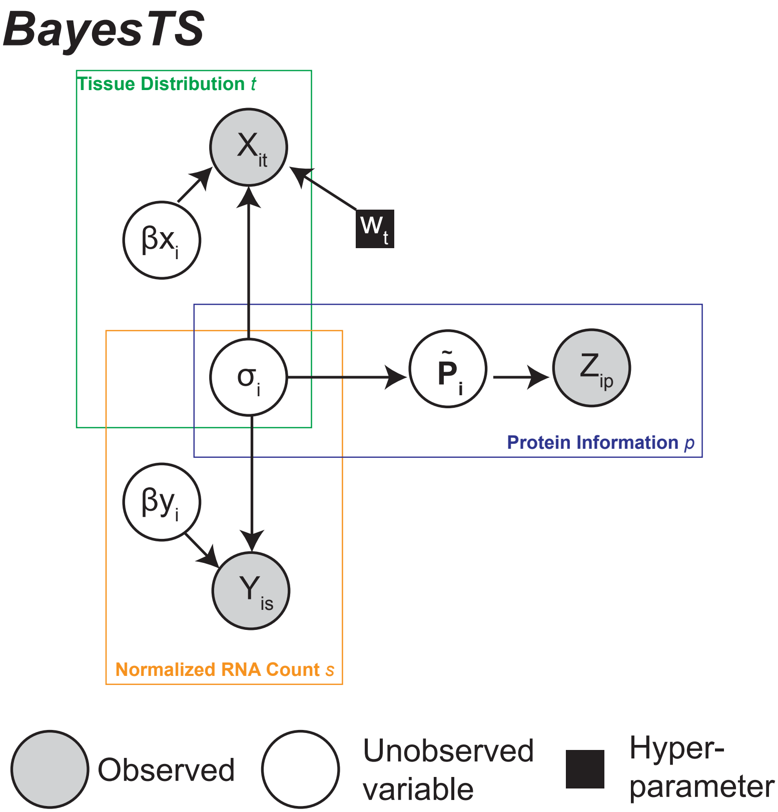

Supplementary Figure 4. The plate notation of the BayesTS model. Each circle represents a random variable in the model shade denotes the observed values and hollow denotes the unobserved values. Black squares represent tunable hyper-parameters. Related to STAR Method.

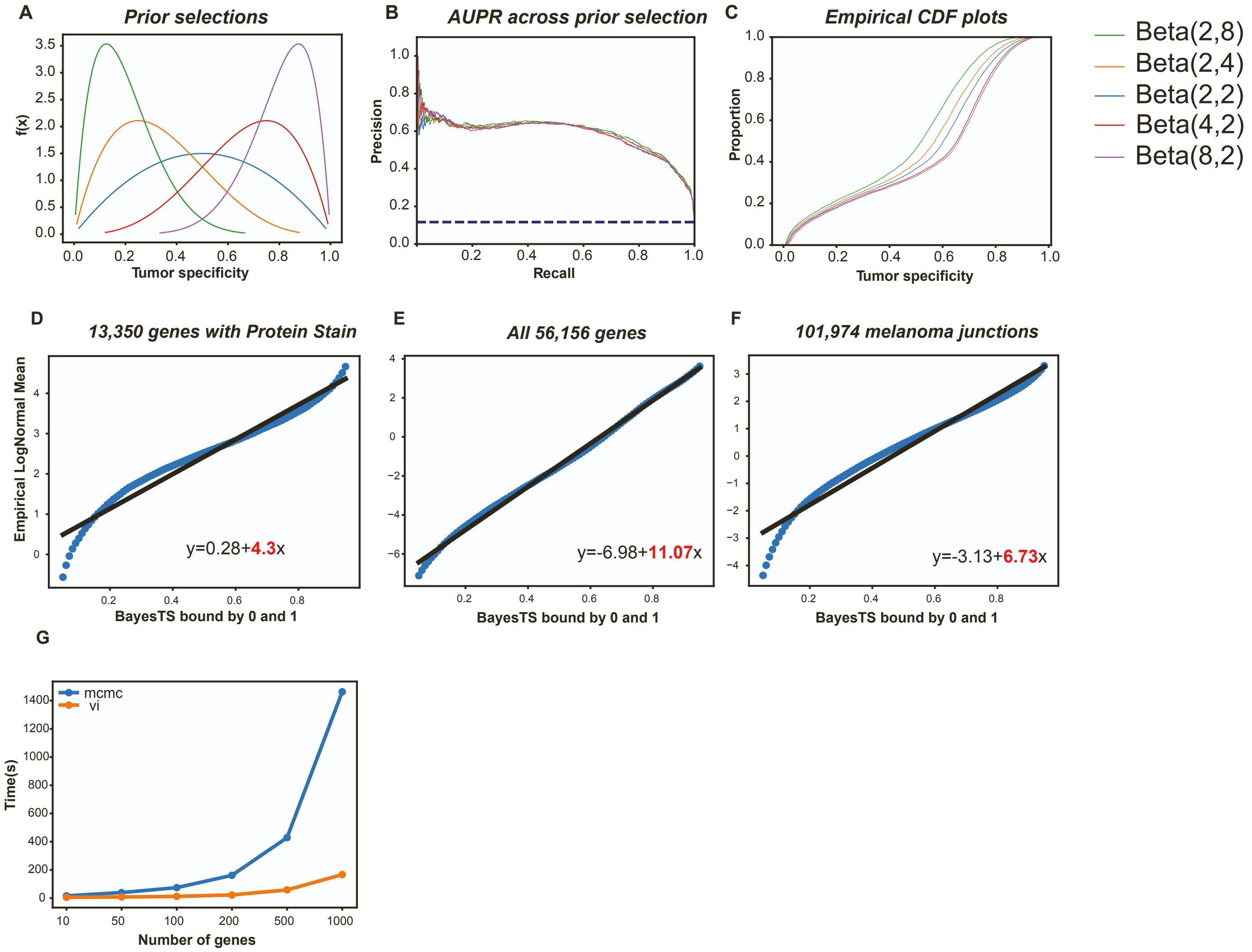

Supplementary Figure 5. Sensitivity analysis, empirical prior determinations and computing performance on different solvers. A) five tested Beta distribution corresponding to decreasing beliefs on tumor-specificity. B) Overall performance on identifying known safe targets across five beta prior. C) eCDF plot of five tested Beta prior on the overall scale of resultant BayesTS score distributions. (D-F) Empirical Bayes approach to derive data-dependent coefficients to account for magnitude difference between sigma and the mean of lognormal distribution to model normalized counts data. (G) Speed comparison on a simplified model and subsampled gene list on pymc5 implemented NUTs MCMC and VI approach. Related to STAR Method and Figure 3.
